# Cross-species transcriptomic evidence for peripheral–central immune crosstalk in atopic dermatitis

**DOI:** 10.3389/fimmu.2026.1790710

**Published:** 2026-06-26

**Authors:** Wenyu Dai, Shengjie Xue, Xin Huang, Xuan Long, Lou Lou, Bolun Wang, Haishan Wu, Jieyue Liao

**Affiliations:** 1 Department of Dermatology, Second Xiangya Hospital, Hunan Key Laboratory of Medical Epigenomics, Clinical Medical Research Center of Major Skin Diseases and Skin Health of Hunan Province, Central South University, Changsha, Hunan, China; 2 Department of Psychiatry, National Clinical Research Center for Mental Disorders, National Center for Mental Disorders, and China National Technology Institute on Mental Disorders, The Second Xiangya Hospital of Central South University, Changsha, Hunan, China; 3 Department of Psychiatry, Guangdong Provincial People’s Hospital, Guangzhou, Guangdong, China; 4 Scientific Research Program of Hunan Provincial Health Commission, Department of Radiology, Clinical Research Center for Medical Imaging in Hunan Province, The Second Xiangya Hospital of Central South University, Changsha, Hunan, China

**Keywords:** atopic dermatitis, functional magnetic resonance imaging, neuroimaging transcriptomics, neuroinflammation, RNA sequencing

## Abstract

**Background:**

Atopic dermatitis (AD) is characterized by peripheral inflammation and intense pruritus. While itch-induced brain activation in AD is documented, our previous work revealed aberrant resting-state activation in the left superior frontal gyrus (LSFG). However, whether this central dysfunction is linked to peripheral immune status remains unclear.

**Methods:**

We integrated neuroimaging transcriptomics based on resting-state functional MRI data from AD patients (n=19) and healthy controls (n=36) with transcriptomic profiling and experimental validation in MC903-induced AD mouse models. Imaging transcriptomics was applied to identify genes associated with abnormal left superior frontal gyrus (LSFG) activation. T follicular helper 13-conditional knockout (Tfh13-cKO) mice were used to investigate whether dampening peripheral inflammation affects CNS neuroinflammation. RNA sequencing, flow cytometry, histology, and RT-qPCR were employed for mechanistic validation.

**Results:**

Neuroimaging transcriptomics revealed that the spatial pattern of aberrant LSFG activation in AD patients was significantly correlated with the expression maps of astrocyte- and microglia-related genes, enhanced inflammatory signaling and dysregulation of dopaminergic and GABAergic neurotransmission according to Allen Human Brain Atlas. Interleukin family members (IL13RA1, IL17RD, IL33) also showed strong positive correlations with LSFG imaging phenotypes. In AD mice, the prefrontal cortex exhibited a pronounced neuroinflammatory phenotype with elevated glial markers (*Gfap, Aif1*) and pro-inflammatory mediators (*Tnf, Il6, Cxcl10*), accompanied by transcriptomic signatures indicative of impaired synaptic plasticity. Notably, Tfh13-cKO AD mice with attenuated peripheral inflammation (reduced IgE, decreased effector T cells and germinal center B cells) displayed significantly alleviated central neuroinflammation, downregulated interferon-alpha response, and restored expression of synaptic plasticity-related genes.

**Conclusion:**

These findings suggest that chronic peripheral inflammation may be associated with neuroinflammation and neurotransmitter imbalance centered in the LSFG and prefrontal cortex, contributing to specific brain activation patterns in AD patients. This study uncovers a novel peripheral-central immune interaction mechanism in AD and provides new insights for developing neuroimmune-targeted therapeutic strategies.

## 1 Introduction

Atopic dermatitis (AD) is a chronic inflammatory skin disorder characterized by dry skin, disseminated eczematous lesions, and persistent pruritus (1, 2). During AD pathogenesis, the immune and nervous systems interact to form a complex regulatory network that perpetuates inflammation and contributes to disease chronicity. At the cutaneous level, Th2-related cytokines such as IL-4, IL-13, and TSLP directly communicate with sensory neurons to induce itch, thereby initiating an itch–scratch cycle that exacerbates skin inflammation (3, 4). Conversely, sensory neurons release neuropeptides (e.g., CGRP, SP, VIP) that act on dendritic cells, mast cells, and other immune cells to modulate cytokine production and reciprocally regulate type II skin inflammation (5–8). At the spinal level, studies by Shiratori-Hayashi, Koga et al. and Liu, Han et al. demonstrated that activation of spinal astrocytes sustains chronic itch in AD or contact dermatitis by releasing LCN2, which acts on GRPR-expressing dorsal horn neurons, thereby indirectly prolonging cutaneous inflammation (9, 10). Despite these insights, the relationship between the central nervous system (CNS) and peripheral immune responses in AD remains insufficiently understood.

In recent years, with advances in neuroscience and cross-disciplinary approaches, increasing attention has been directed toward CNS activation patterns in diverse diseases. Several studies have investigated brain imaging alterations in AD. Gudrun Schneider (11) and Y. Ishiuji et al. (12) reported that, during histamine-induced acute itch, AD patients exhibit abnormal activation in prefrontal regions (including dorsolateral prefrontal cortex and anterior cingulate cortex) and the posterior cingulate cortex—collectively known as the “itch matrix.” Our previous work further revealed abnormal resting-state activation of the left superior frontal gyrus (LSFG) in AD patients compared with healthy controls, suggesting intrinsic functional alterations of the AD brain (13). Notably, LSFG functional connectivity with other abnormally activated regions was negatively correlated with peripheral disease severity, indicating a potential link between peripheral inflammation and CNS functional or structural changes. This observation is consistent with Li et al., who reported associations between circulating pro-inflammatory cytokines and reduced gray matter volume in AD patients (14).

The impact of peripheral immune inflammation on brain function and structure has long been debated, with most evidence arising from animal models. Current studies suggest that peripheral inflammation can induce CNS neuroinflammation, leading to altered neural activity (15). Systemic LPS administration is known to activate microglia and trigger neuroinflammation in mice, forming a classical model of CNS immune activation (16). Thomson et al. further demonstrated that multiple Toll-like receptor–dependent inflammatory models—including LPS injection and topical imiquimod—upregulate type I interferon signaling in the mouse brain, suggesting potential transcriptional reprogramming of the CNS in response to peripheral inflammation (17). However, attempts to model human CNS responses using animal brains are inherently limited by species differences.

To elucidate the cellular and molecular mechanisms underlying the abnormal CNS activation patterns observed in AD patients, we employed imaging transcriptomics—an emerging technique that integrates spatial gene expression profiles with neuroimaging phenotypes derived from brain-wide transcriptional atlases (18). This approach has been increasingly applied to investigate neurodevelopmental, neurodegenerative, and psychiatric disorders (19). Using imaging transcriptomics, we found that abnormal LSFG activation in AD patients was associated with astrocyte and microglial activation and upregulation of inflammatory pathways, suggesting a neuroinflammatory basis for the functional abnormalities. Moreover, several interleukin family members showed strong positive correlations with LSFG imaging phenotypes, further supporting the presence of immune activation within the AD brain. Complementary MRI–nuclear imaging cross-modal analyses revealed dysregulation of dopaminergic, GABAergic, and noradrenergic neurotransmitter systems in the LSFG, indicating that neuroinflammation and neurotransmitter imbalance may jointly contribute to the abnormal CNS activation patterns in AD.

To validate these human findings, we conducted transcriptomic analyses of the prefrontal cortex—the anatomical and functional homolog of the human LSFG—in an AD mouse model and observed pronounced neuroinflammatory signatures and synaptic dysfunction. High-affinity IgE is a hallmark of allergic inflammation, and Tfh13 cells are critical drivers of this process. Given the essential role of high-affinity IgE in AD, we further employed T follicular helper 13–conditional knockout (Tfh13-cKO) mice to induce AD model, which exhibit impaired IgE production and attenuated peripheral allergic responses (20). Transcriptomic and experimental analyses demonstrated that reduced peripheral inflammation markedly ameliorated CNS neuroinflammation, providing mechanistic evidence linking peripheral immune activity to central neuroimmune alterations in AD.

Overall, this study delineates the multidimensional mechanisms underlying abnormal CNS activation in AD, including glia-driven neuroinflammation, widespread neurotransmitter dysregulation, and substantial peripheral-to-central immune crosstalk. These findings highlight the immune–neural axis as a potential target for precision therapeutics in AD.

## 2 Materials and methods

### 2.1 Subjects

Nineteen AD participants (12M/7F, mean age, 30.21 ± 2.59 years) were enrolled from the Department of Dermatology at the Second Xiangya Hospital of Central South University, during 2021–2023. Participants aged between 18 and 60 years with a confirmed diagnosis of AD based on the international criteria of Hanifin and Rajka (21) and Severity Scoring of Atopic Dermatitis (SCORAD) score exceeding 25 were included. For a detailed description of the eligibility and exclusion criteria, please refer to Supplementary Material (See **Supplementary Method 1**).

Thirty-six HCs (13M/23F, mean age, 20.25 ± 0.20 years) were recruited from the community, hospital staff, and schools. None had any skin or systemic diseases, nor any history of psychiatric or neurological disorders. The demographic and clinical characteristics of the participants are summarized in **Table 1**.

**Table 1:** Demographics and clinical characteristics of the participants.

Characteristics	Healthy control	AD	*P* value
Gender			0.055^a^
male, n (%)	13 (36)	12 (63)	
female, n (%)	23 (64)	7 (37)	
Age, Median (IQR)	20 (1)	27(14)	P<0.001^d^ ^*^
Education			0.011^b^ ^*^
Primary Education or below, n (%)	0	0	
Secondary Education, n (%)	0	4 (21)	
Higher Education, n (%)	36 (100)	15 (79)	
Disease duration, M (SD)	NA	12.7 (2.0)	NA
Height, m, M (SD)	1.67 (0.01)	1.68 (0.02)	0.665^c^
Weight, kg, M (SD)	60.9 (2.4)	61.6 (2.6)	0.861^c^
BMI, kg/m2, M (SD)	21.63 (0.64)	21.77 (0.77)	0.892^c^
Mean FD Jenkinson, Median (IQR)	0.08 (0.04)	0.11 (0.07)	0.002^d^ ^*^

a The P value was obtained using a two-tailed Pearson chi-square test.

b The P value was obtained using Fisher’s exact test.

c The P value was obtained using a two-sample two-tailed t test.

d The P value was obtained using a Mann-Whitney U test.

M, mean; SD, standard deviation; IQR, inter-quartile range; BMI, body mass index; FD, frame displacement.

*P < 0.05.

The Institutional Review Boards of the Second Xiangya Hospital of Central South University reviewed and approved the design of this study. Written informed consents were obtained from all participants.

### 2.2 Transcriptome-neuroimaging spatial correlation and gene category enrichment analysis

Workflow of MRI scanning, MRI Data processing and neuroimaging features calculation procedure has been detailed in previous publications (13) (See **Supplementary Methods 3–6**). After collecting and preprocessing functional MRI data, neuroimaging features including degree centrality (DC) and functional connectivity (FC) were calculated (See **Supplementary Method 7**). Then we mapped results from DC and FC analysis using the Desikan-Killiany atlas and thus creating the Z map (22). Using the Imaging Transcriptomics toolbox (23), we applied Spearman correlation to establish the relationship between results from neuroimaging features and preprocessed gene expression measurements for all 15633 genes (See **Supplementary Method 7**) to rank gene correlations. To gain insights into the biological significance of our findings, we further performed Gene set enrichment analyses (GSEA) using the “Lake” brain cell-type gene set, Hallmark gene set, Kyoto Encyclopedia of Genes and Genomes (KEGG) pathways and Gene Ontology (GO) cellular component.

### 2.3 MRI-nuclear imaging cross-modal correlation analysis

JuSpace (https://github.com/juryxy/JuSpace) was adopted to investigate the spatial correlations of the t map with nuclear imaging derived measures covering various neurotransmitter systems including dopamine, serotonin, glutamate, GABA, acetylcholine, opioid, cannabinoid, noradrenaline, and fluorodopa (see **Supplementary Method 8**; **Supplementary Table 4**) (24).

### 2.4 Generation of IL-13^cre^ Bcl6^flox/flox^ (Tfh13-cKO) mice

To generate IL-13^cre^ Bcl6^flox/flox^ mice (Tfh13 cell–specific Bcl6 conditional knockout), we first established two parental strains: C57BL/6N-IL-13-e (IRES-iCre) and C57BL/6N- Bcl6^flox/flox^.

For the IL-13-e (IRES-iCre) line, an IRES-iCre expression cassette was inserted immediately downstream of the Il13 stop codon through CRISPR/Cas9-mediated homologous recombination. Cas9 mRNA and gRNA were synthesized by *in vitro* transcription, and a donor vector containing a 3.0 kb 5′ homologous arm, IRES-iCre, and a 3.0 kb 3′ homologous arm was constructed using the In-Fusion cloning method. The Cas9 mRNA, gRNA, and donor vector were co-microinjected into C57BL/6N fertilized eggs to generate F0 founders. Positive F0 mice identified by PCR and sequencing were crossed with C57BL/6N mice to obtain F1 heterozygotes carrying the Il13-IRES-iCre allele.

For the Bcl6^flox/flox^ line, exons 5–6 (between exon 3 containing the start codon ATG and exon 10 containing the stop codon TGA; transcript ID: ENSMUST00000023151) were selected as the conditional knockout region. Deletion of this region results in loss of Bcl6 gene function. A targeting vector was constructed using BAC clone RP23-33K13 as a template to amplify homologous arms and the cKO region by PCR. Cas9 mRNA, gRNA, and the targeting vector were co-injected into fertilized C57BL/6N zygotes to obtain founder mice by homologous recombination. Offspring were genotyped by PCR and confirmed by sequencing. Finally, IL-13^cre^ mice were crossed with Bcl6^flox/flox^ mice to generate IL-13^cre^ Bcl6^flox/flox^ (Tfh13-cKO) mice for subsequent experiments.

### 2.5 Animal model construction and transcriptional analysis of mouse brain

#### 2.5.1 Animals, brain tissue sampling and RNA sequencing

Where indicated, 6-week-old C57BL/6N IL-13^cre^ Bcl6^flox/flox^ mice were utilized to construct a Tfh13-cKO model in this study. Both wild-type mice and Tfh13-cKO mice received a daily topical application of 50ul 1α,25-(OH)_2_D_3_ Low-Calcemic Analog (0.005% Calcipotriene topical solution, LEO company) to the right ear for two weeks to induce an AD phenotype. At sacrifice, the brain tissues were harvested, and the prefrontal cortex were then isolated. All the tissues were freshly frozen and stored at -80 °C for subsequent RNA Extraction, library construction, sequencing (See **Supplementary Method 9**) and transcriptional analysis.

#### 2.5.2 Differential expression analysis and enrichment analysis

RNAs differential expression analysis between PFC of two different groups was performed via DESeq2 (25) (See **Supplementary Method 10**). Next, we applied functional enrichment analysis on the identified DEGs based on the Database for Annotation, Visualization and Integrated Discovery (DAVID) (26, 27). Additionally, GSEA was conducted based on transcriptional data from PFC of AD mice and Tfh13-cKO AD mice (See **Supplementary Method 11**).

### 2.6 Flow cytometry identification

Draining lympho-nodes of right ear and spleen were collected, primary cells were isolated using Falcon 40um cell strainer (CORRNING, USA). To examine the expression of surface markers, cells were incubated with1:200 FcR blocking reagent (Miltenyi, Germany) for 10 min followed by primary antibodies on ice in the dark for 30 min. The antibodies used for surface marker analysis included anti-mouse CD4-APCCY7 (BD Pharmingen, USA, 1:400), CXCR5 Biotin rat anti-mouse antibody, PD-1-APC (BD Pharmingen, USA, 1:400), PE goat anti-rat antibody (1:400) for Tfh cells. B220-FITC (BD Pharmingen, USA, 1:400), CD44-PECY7 (Biolegend, USA, 1:400), CD4-PerCP-Cy5.5 (BD Pharmingen, USA, 1:400), FAS-PE (BD Pharmingen, USA, 1:400), GL-7 (BD Pharmingen, USA, 1:400), CD62L-APCCY7 (BD Pharmingen, USA, 1:400) for effector and memory T cells and GC B cells. CD19-FITC (BD Pharmingen, USA, 1:400), CD3-APCCY7 (BD Pharmingen, USA, 1:400), CD4-PECY7 (BD Pharmingen, USA, 1:400), CD8 PerCP-Cy5.5 (BD Pharmingen, USA, 1:400), NK1.1-APC (BD Pharmingen, USA) for T cells, B cells and NK cells. Data were acquired by flow cytometry (BD, Canto II, USA) and analyzed using FlowJo (Tree Star, USA).

### 2.7 Histological evaluation

Mouse ear samples were collected, wax sealed and cut into sections. Skin sections of the ear were stained with hematoxylin and eosin (H&E). Histological evaluation was performed on each slide.

### 2.8 IgE measurement

Mouse serum was collected on day 14, serum IgE was measured by mouse IgE Elisa kit (Zcibio Company) using Rayto RT-6100.

### 2.9 Real-time quantitative PCR

RNA was extracted using the TRIzol method. Reverse transcription was performed using Evo M-MLV RT Reaction Mix (Accurate Biology, China) to synthesize first-strand complementary DNA (cDNA) from 1 ng of total RNA. Real-time quantitative PCR was conducted on an ABI 7900 Real-Time PCR System. Relative gene expression levels were calculated using the 2^−ΔΔCt^ method, with Gapdh serving as the internal control for normalization. The primers used in this study are listed in **Supplementary Table 6**.

### 2.10 Immunofluorescence

Initially, 20-μm-thick coronal brain sections were rinsed three times in PBS for 5 minutes each to ensure equilibration. To prevent non-specific binding and enhance permeability, the slices were pre-incubated for 1 hour at room temperature in a blocking buffer (0.3% Triton X-100 and 10% goat serum in PBS). Subsequently, the tissue was subjected to overnight incubation at 4 °C with the primary antibody against Iba1 (FUJIFILM Wako, 1:400). On the following day, the sections were labeled with Alexa Fluor-conjugated secondary antibodies (Thermo Fisher) for 1 hour at ambient temperature, strictly protected from light. After three additional 5-minute washes in PBS, the specimens were mounted onto glass slides using a suitable mounting medium and coverslipped. Representative fluorescence images were captured via PanoBrain automated brain mapping and analysis system (PanoBrain, Meca Scientific, China).

### 2.11 Statistical analysis

Statistical analyses were performed using R (Version 4.4.1), GraphPad Prism 10, and the DESeq2 package for transcriptomic differential expression. The normality of data distribution was evaluated by the Shapiro-Wilk test. For comparisons between two groups, unpaired Student’s t-tests were used for normally distributed data, while the Mann-Whitney U test was applied for non-normally distributed variables. Difference with P value < 0.05 was considered statistically significant.

## 3 Result

### 3.1 Neuroimaging transcriptomics unveils the involvement of neuroinflammation in LSFG in AD patients

**Figure 1:**
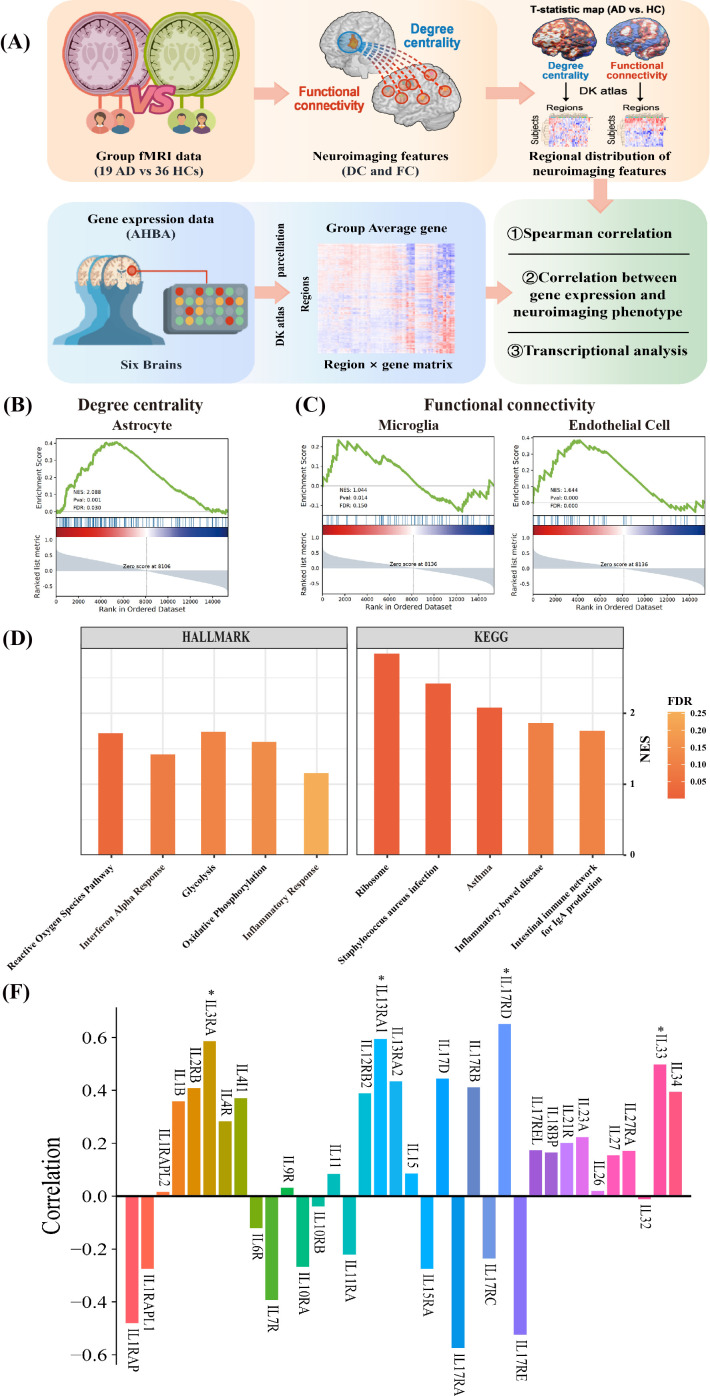
Neuroimaging transcriptomics unveils the involvement of neuroinflammation in LSFG in AD patients. **(A)** Workflow chart of neuroimaging transcriptomic analysis. **(B)** GSEA using the Lake cell set of DC difference in AD patients compared to healthy controls. A significant association between DC differences and astrocytes in AD patients was identified (NES = 2.09, p = 0.001, FDR = 0.03). **(C)** GSEA using the Lake cell set of functional connectivity in AD patients compared to healthy controls. A significant association between FC differences, microglial and endothelial cells in AD patients was identified. **(D)** Terms from the GSEA-Hallmark and GSEA-KEGG pathways enrichment analysis of genes correlated with DC differences between AD patients and HCs. Only terms with |NES| > 1, p-value < 0.05 and FDR q-value < 0.25 were considered significant. **(E)** Correlations between degree centrality and the interleukin family members based on neuroimaging transcriptional profile of LSFG. “*” means adjusted P value < 0.05. (For GSEA and cell-type enrichment analyses, gene sets with an absolute Normalized Enrichment Score (NES) > 1 and a False Discovery Rate (FDR) < 0.25 were considered significantly enriched. Regarding the spatial transcriptomic correlation between DC values and target genes, an adjusted P-value < 0.05 was defined as statistically significant).

**Figure 2:**
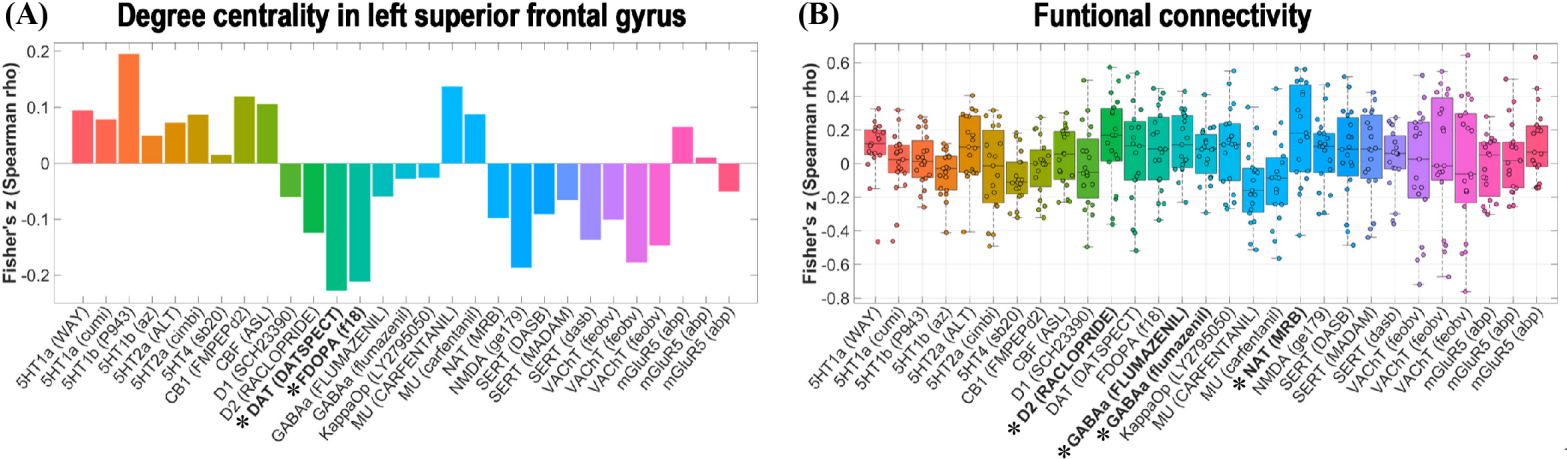
MRI-nuclear imaging cross-modal correlations indicate the dysregulation of neurotransmitter system in LSFG and its aberrantly functionally connected brain regions of AD patients. **(A)** Barplots of cross-modal correlations between receptor systems and DC differences in the LSFG in AD patients compared to HCs. **(B)** Barplots of cross-modal correlations between receptor systems and FC differences in AD patients compared to HCs. (Neurotransmitter system components shown in bold represent those significantly correlated with differences in DC values or functional connectivity (FC) abnormalities between AD patients and healthy controls; “*” indicate an adjusted P-value < 0.05).

Based on the expression matrix associated with the aberrant neuroimaging phenotype of the left superior frontal gyrus in AD patients derived from neuroimaging–transcriptomics analysis, further investigations were performed (See **Figure 1A**). Cell type enrichment analysis indicated a significant association between DC differences and astrocytes in AD patients compared to HCs (normalized enrichment score (NES) = 2.09, p = 0.001, false discovery rate (FDR) = 0.03, **Figure 1B**). The same methodology was applied to the FC difference T-maps between AD patients and HCs. It was indicated that FC differences were significantly associated with upregulation in microglia (NES > 1, p = 0.014, FDR = 0.015, **Figure 1C**) and endothelial cells (NES > 1, p < 0.001, FDR< 0.001, **Figure 2B**). Interestingly, all three cell types are considered key participants or initiators in neuroinflammatory processes.

Gene set enrichment analyses (GSEA) using Analysis with Hallmark gene sets showed that DC differences in AD patient’s brain were mainly associated with pathways like “inflammatory response” and “interferon alpha response” (see **Figure 1D**; **Supplementary Table 1**), which may indicate upregulation of inflammatory pathway. Analysis with Kyoto Encyclopedia of Genes and Genomes (KEGG) gene sets implied the relationship between DC differences and pathways like “asthma” and “staphylococcus infection” (see **Figure 1E** and **Supplementary Table 3**).

Although members of the interleukin family are traditionally regarded as key mediators of peripheral inflammation, emerging evidence indicates that they also play important roles within the central nervous system, including the regulation of neuroinflammation and synaptic plasticity (28–31).To further explore potential alterations in the immune status of the brain in patients with atopic dermatitis, we examined the association between imaging phenotypes and the interleukin family based on brain imaging transcriptomic data, using the left superior frontal gyrus (LSFG) as the seed region. Compared with healthy controls, AD patients showed positive correlations between LSFG imaging phenotypes and the expression levels of IL3RA (r=0.586, padj<0.001), IL13RA1 (r=0.594, padj<0.001), IL17RD (r=0.651 padj=0.014), and IL33 (r=0.498, padj=0.014), suggesting upregulation of these interleukin family members in this brain region of AD patients (See **Figure 1F**).

### 3.2 MRI-nuclear imaging cross-modal correlations indicate the dysregulation of neurotransmitter system in LSFG of AD patients

Cross-modal correlations revealed associations between DC differences in the LSFG and the dopaminergic system as well as between FC differences and the dopaminergic system, GABAergic and noradrenaline system (see **Figure 2**; **Supplementary Table 4**). Specifically, the DC differences in the LSFG were negatively related to dopamine transporter (DAT; r = -0.227, P = 0.009) and 6- (18)F-fluoro-L-dopa (FDOPA; r = -0.212, P = 0.021) (See **Figure 2A**). And FC differences in AD compared to HCs were positively related to dopamine receptor D2 (r = 0.134, P = 0.028), GABA sub-type A receptor (GABAA; r = 0.122, P = 0.016) and noradrenaline transporter (NAT; r = 0.189, P = 0.002) (See **Figure 2B**).

### 3.3 Integrated transcriptomic and experimental analyses recapitulate an augmented neuroinflammatory phenotype in AD mouse models

**Figure 3:**
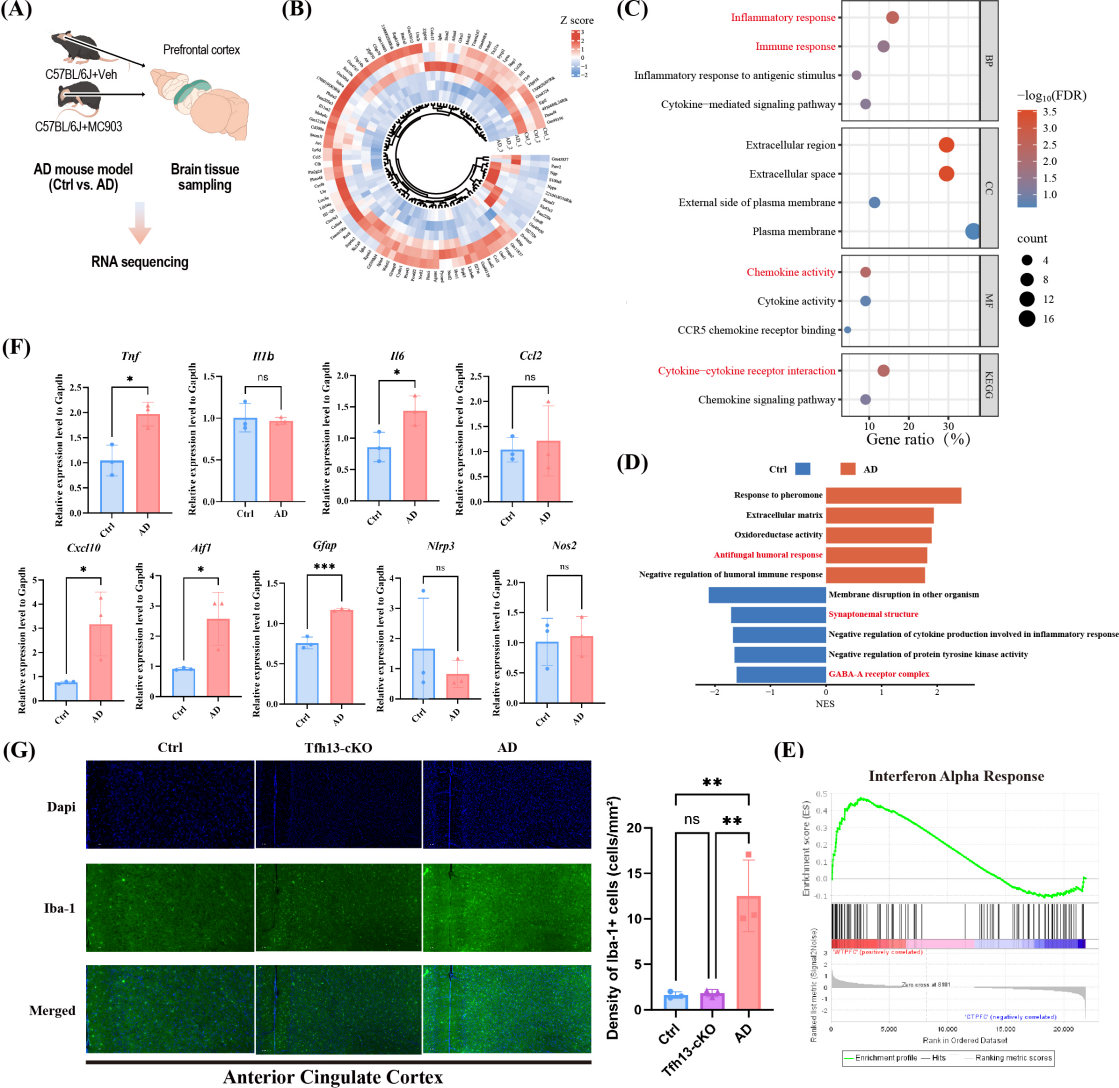
RNA-seq profiling and RT-qPCR validation reveal pronounced neuroinflammatory responses and downregulation of synaptic plasticity in the prefrontal cortex of AD mice. **(A)** Brain tissue sampling of the prefrontal cortex from control and AD mice. **(B)** Circular heatmap showing the expression profiles of differentially expressed genes (DEGs). Each spoke represents an individual gene, and each concentric ring represents a sample from either the control group or the AD group. Gene expression levels are visualized as Z-scores calculated by row-scaling the normalized counts. The color scale indicates the relative expression level, where red (positive Z-score) represents up-regulation and blue (negative Z-score) represents down-regulation relative to the mean. **(C)** Functional analysis of DEGs between mice of control group and those of AD group. Pathways highlighted in red are inflammation-related. **(D)** GSEA-GO analysis of expression matrix from prefrontal cortex of control group and AD group. GO terms upregulated in each group were displayed. **(E)** GSEA-Hallmark pathway analysis reveals upregulation of interferon alpha response in PFC of AD mice. **(F)** RT-qPCR validation of neuroinflammatory and glial activation markers in the PFC of AD mice. **(G)** Representative images of DAPI (blue, nuclei) and Iba-1 (green, microglia) staining in the ACC of Ctrl, Tfh13-cKO and AD mice (left, scale bar = 100 μm). The bar graph (right) shows the density of Iba-1+ microglia (cells/mm^2^) in the ACC. A significant elevation in microglial density was observed in the AD group compared to the Ctrl and Tfh13-cKOgroup. Data are presented as mean ± SEM (n = 3 per group). Statistical significance was determined using an unpaired two-tailed Student’s t-test; P < 0.05 was considered significant. *P < 0.05, **P < 0.01, ***P< 0.001; NS, not significant.

In this study, we used the brain transcriptome data of AD mice and ctrl mice (see **Figure 3A**). After normalizing the read count data, the DESeq2 package was employed for differential gene expression analysis. In PFC, we identified 74 differentially expressed genes (13 upregulated, 88 downregulated in AD mice) between ctrl mice and AD mice (see **Figure 3B**). Then we performed GO and KEGG enrichment analysis based on these DEGs. As depicted in the diagram, in PFC, the differentially expressed genes were primarily associated with inflammation related biological processes such as “inflammatory response”, “immune response”, “chemokine activity” and “cytokine-cytokine receptor response”. (see **Figure 3C**). Meanwhile, GSEA analysis using GO gene set indicate the down regulation of gene set related to “synaptonemal structure” and “GABA-A receptor complex”, which were mainly associated with synaptic plasticity (see **Figure 3D**). Similarly, consistent with human imaging-transcriptomics data, we observed an upregulation of the “interferon alpha response” pathway in AD mice. Notably, the leading-edge analysis revealed that this enrichment was primarily driven by a core set of interferon-associated genes, including interferon-stimulated genes (ISGs) such as *Isg15, Ifit3, Oas1a, Stat2, Irf7, Irf9*, and *Usp18*, as well as other interferon-induced mediators like *Ifitm1/2/3, Cxcl10, Bst2*, and *Trim25* (See **Supplementary Table 6**). We further validated the expression of neuroinflammation-related genes using RT-qPCR. The results showed that the pro-inflammatory cytokines *Tnf* (encoding TNF-α) and *Il6* were significantly upregulated in AD mice, whereas *Il1b* remained unchanged. Among chemokines, *Cxcl10* was markedly increased, while *Ccl2* showed no significant difference. Notably, the mRNA levels of Aif1 (encoding the microglial marker Iba-1) and *Gfap* (an astrocytic marker) were both significantly elevated, indicating glial activation. In contrast, the inflammasome component *Nlrp3* and nitric oxide synthase *Nos2* did not exhibit significant changes (see **Figure 3E**). To further corroborate the neuroinflammatory landscape in the PFC, we performed immunofluorescence staining on brain sections from Ctrl, Tfh13-conditional knockout (Tfh13-cKO) and AD mice. Given that the anterior cingulate cortex (ACC) is a critical node within the PFC involved in pruritus processing, we quantified Iba-1+ microglia in this region. Our results revealed a significant increase in the density of Iba-1+ microglia in the ACC of AD mice compared to Ctrl and Tfh13-cKO mice, suggesting microglial proliferation or infiltration. Moreover, microglia in AD mice exhibited activation-associated morphological changes, characterized by enlarged somata and shortened, retracted branches (see **Figure 3G**). These histological findings are consistent with the upregulated Aif1 expression observed via RT-qPCR, collectively confirming robust microglial activation in the ACC of AD models. These results confirm the presence of enhanced neuroinflammatory responses in the PFC of AD mice.

### 3.4 Peripheral immunological changes in AD mice may influence neuroinflammation signature in prefrontal cortex

Tfh13 cells are a subset of T follicular helper cells that have been demonstrated in multiple studies to play a critical role in the production of high-affinity IgE during allergic inflammation (20). Conditional deletion of Tfh13 cells has been shown to effectively attenuate the severity of allergic inflammatory responses. Considering that topically applied anti-inflammatory agents in mouse models may be systemically absorbed through the skin and cross the blood–brain barrier to act on the central nervous system, we employed Tfh13-cKO mice to establish an atopic dermatitis (AD) model, aiming to investigate whether attenuation of peripheral inflammation could ameliorate the neuroinflammatory phenotype in the prefrontal cortex of AD mice.

#### 3.4.1 Immune cell characterization in mice with Tfh13 cell-conditional knockout

**Figure 4:**
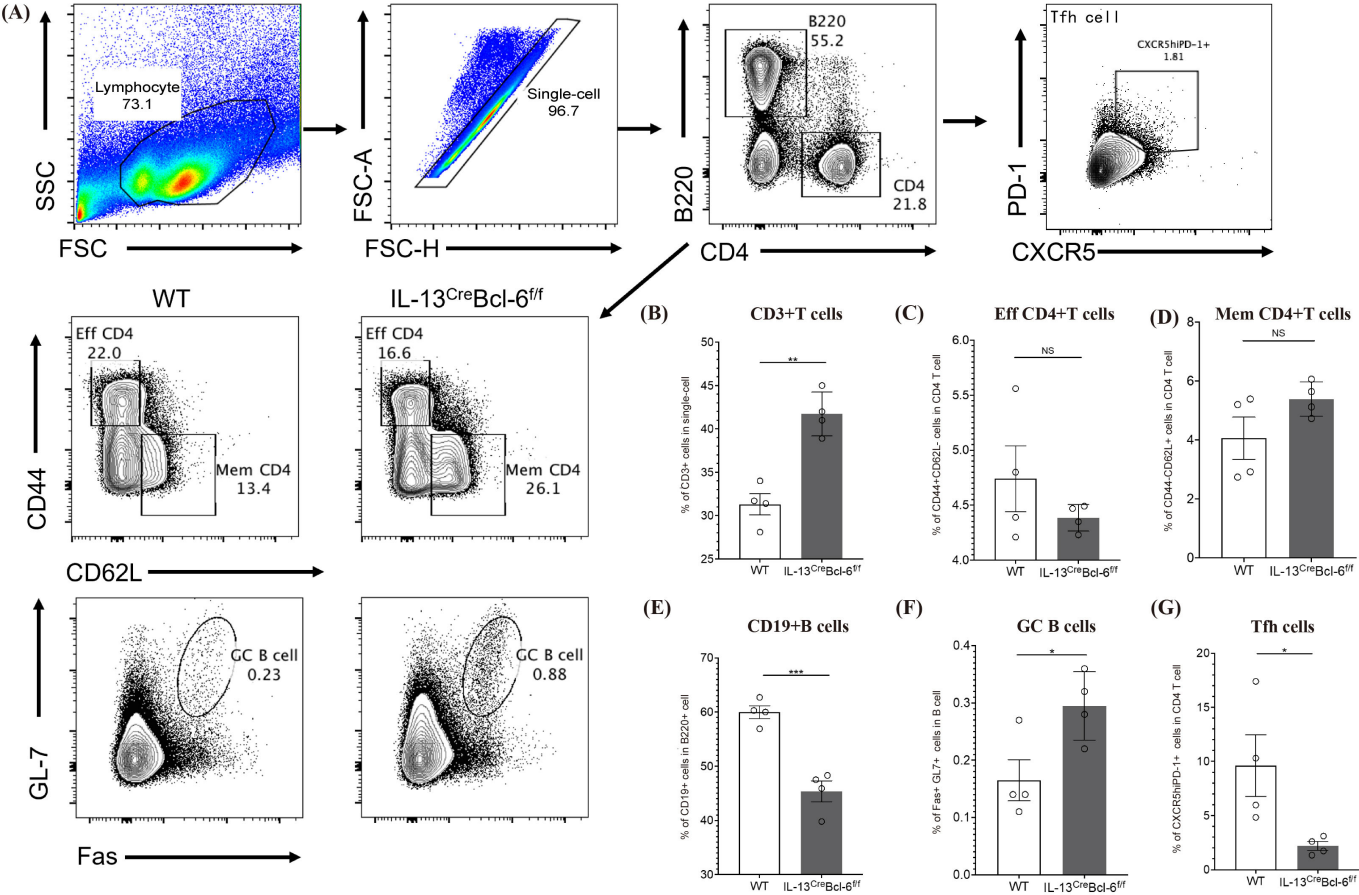
Flow cytometry identification of mice with Tfh13 cell-conditional knockout. **(A)** Gating strategy for the identification of splenic lymphocyte populations. Lymphocytes were gated based on FSC and SSC parameters, followed by single-cell discrimination (FSC-A vs. FSC-H). CD4^+^ T cells and B220^+^ B cells were subsequently identified. Representative plots show effector CD4^+^ T cells (Eff CD4^+^: CD62L^-^CD44^+^), memory CD4^+^ T cells (Mem CD4^+^: CD62L^+^CD44^-^), and germinal center (GC) B cells (B220^+^Fas^+^GL-7^+^) in wild-type AD and Tfh13-cKO AD mice. **(B)** Frequency of CD3+ T cells among total splenocytes. **(C)** Frequency of effector CD4+ T cells (CD44+ CD62L-). **(D)** Frequency of memory CD4+ T cells (CD44- CD62L+) gated on total CD4+ T cells. **(E)** Frequency of germinal center (GC) B cells (Fas+ GL7+) gated on B220+ B cells. **(F)** Serum IgE levels measured by ELISA. **(G)** Frequency of T follicular helper (Tfh) cells (CXCR5hi PD-1+) gated on CD4+ T cells. Data are presented as mean ± SEM (n = 4 per group). Statistical analysis was performed using an unpaired two-tailed Student’s t-test; *P < 0.05, **P < 0.01, ***P < 0.001; NS, not significant.

Flow cytometry was performed to analyze splenic immune cell subsets in wild-type and Tfh13-cKO mice, including total T cells (See **Figure 4B**), effector T cells (**Figure 4C**), memory T cells (See **Figure 4D**), B cells (See **Figure 4E**), germinal center (GC) B cells (See **Figure 4F**), and T follicular helper (Tfh) cells See **Figure 4G**). The results showed a significant increase in total splenic T cells but a marked decrease in Tfh cells in Tfh13-cKO mice compared with wild-type mice. The proportion of memory CD4^+^ T cells exhibited an increasing trend, whereas effector T cells showed a decreasing tendency, suggesting impaired effector T-cell activation following Tfh13 cell depletion. Moreover, the frequency of CD19^+^ B cells was reduced, while GC B-cell reactivity was significantly elevated, indicating a compensatory enhancement of GC B-cell responses due to impaired GC B-cell development after Tfh13 cell knockout.

#### 3.4.2 Deletion of Tfh13 cells can alleviate the onset of AD in mice

**Figure 5:**
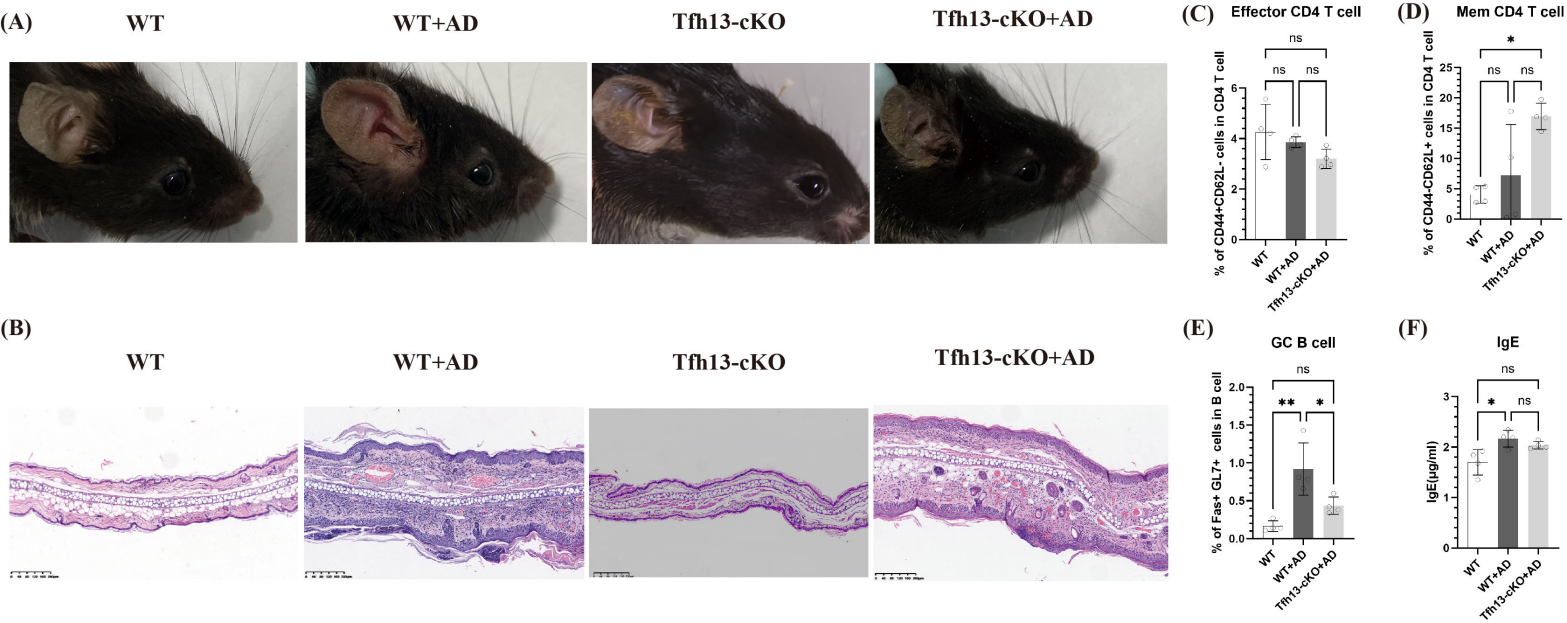
The effect of Tfh13 cell depletion on the development of AD in mice. **(A)** Representative images of the ears from control, wild-type AD, Tfh13-cKO and Tfh13-cKO+AD group mice following 14 days of topical calcipotriol treatment. AD mice with Tfh13-cKO exhibited visibly milder erythema, edema, and scaling compared with wild-type AD mice. **(B)** Representative hematoxylin and eosin (H&E)-stained sections of ear skin showing attenuated epidermal hyperplasia, reduced keratinization, and decreased inflammatory infiltration in Tfh13-cKO AD mice compared with AD mice. Scale bar = 100μm. **(C–E)** Flow cytometric analysis of cervical draining lymph nodes showing that the proportion of effector CD4^+^ T cells (CD4^+^CD62L^-^CD44^+^) was significantly reduced **(C)**, memory CD4^+^ T cells (CD4^+^CD62L^+^CD44^-^) showed an increasing trend [**(D)**, P = 0.0638], and germinal center (GC) B cells (B220^+^Fas^+^GL-7^+^) were significantly decreased **(E)** in Tfh13-cKO mice compared with AD mice. **(F)** Serum IgE levels showed a downward trend in Tfh13-cKO mice compared with AD mice, although the difference was not statistically significant. Data are presented as mean ± SEM (n = 4 per group). Statistical significance was determined using an unpaired two-tailed Student’s t-test; P < 0.05 was considered significant. P < 0.05, P < 0.01; ns, not significant. *P < 0.05, **P < 0.01; NS, not significant..

To induce the AD model, 6-week-old wild-type (WT) and Tfh13-cKO mice were topically treated with calcipotriol (60 mg/day) on the right ear for 14 consecutive days. We then compared ear skin lesions, histopathological changes, and alterations in T and B lymphocytes within the draining lymph nodes and spleen across four groups: WT mice, WT AD mice, Tfh13-cKO mice, and Tfh13-cKO AD mice. Tfh13-cKO AD mice exhibited milder ear skin lesions compared with AD mice, characterized by reduced edema, scaling, dryness, and desquamation (see **Figure 5A**). Histological analysis of ear skin by H&E staining revealed that Tfh13-cKO AD mice had thinner epidermal and dermal layers, attenuated hyperkeratosis, and decreased inflammatory cell infiltration relative to AD mice (see **Figure 5B**). Serum IgE levels in Tfh13-cKO AD mice also showed a decreasing trend compared with AD mice (see **Figure 5F**). These findings suggest that Tfh13 cell depletion may alleviate AD skin lesions, potentially through reduction of serum IgE levels. Flow cytometric analysis further revealed that, compared with AD mice, Tfh13-cKO AD mice exhibited a significant decrease in effector CD4^+^ T cells (CD4^+^B220^-^CD44^+^CD62L^-^) and GC B cells (B220^+^Fas^+^GL-7^+^) in the cervical draining lymph nodes (**Figures 5C, E**, P < 0.05), whereas memory CD4^+^ T cells (CD4^+^B220^-^CD44^-^CD62L^+^) showed an increasing trend (P = 0.0638) (see **Figure 5D**). Collectively, these results indicate that Tfh13 cells play a critical role in the pathogenesis of AD, and their deletion may attenuate disease by reducing effector T-cell activation and the number of germinal center B cells in local lymph nodes, thereby decreasing IgE production and alleviating AD manifestations in mice.

#### 3.4.3 The prefrontal cortex of Tfh13-cKO AD mice exhibited attenuated neuroinflammation and enhanced synaptic plasticity

**Figure 6:**
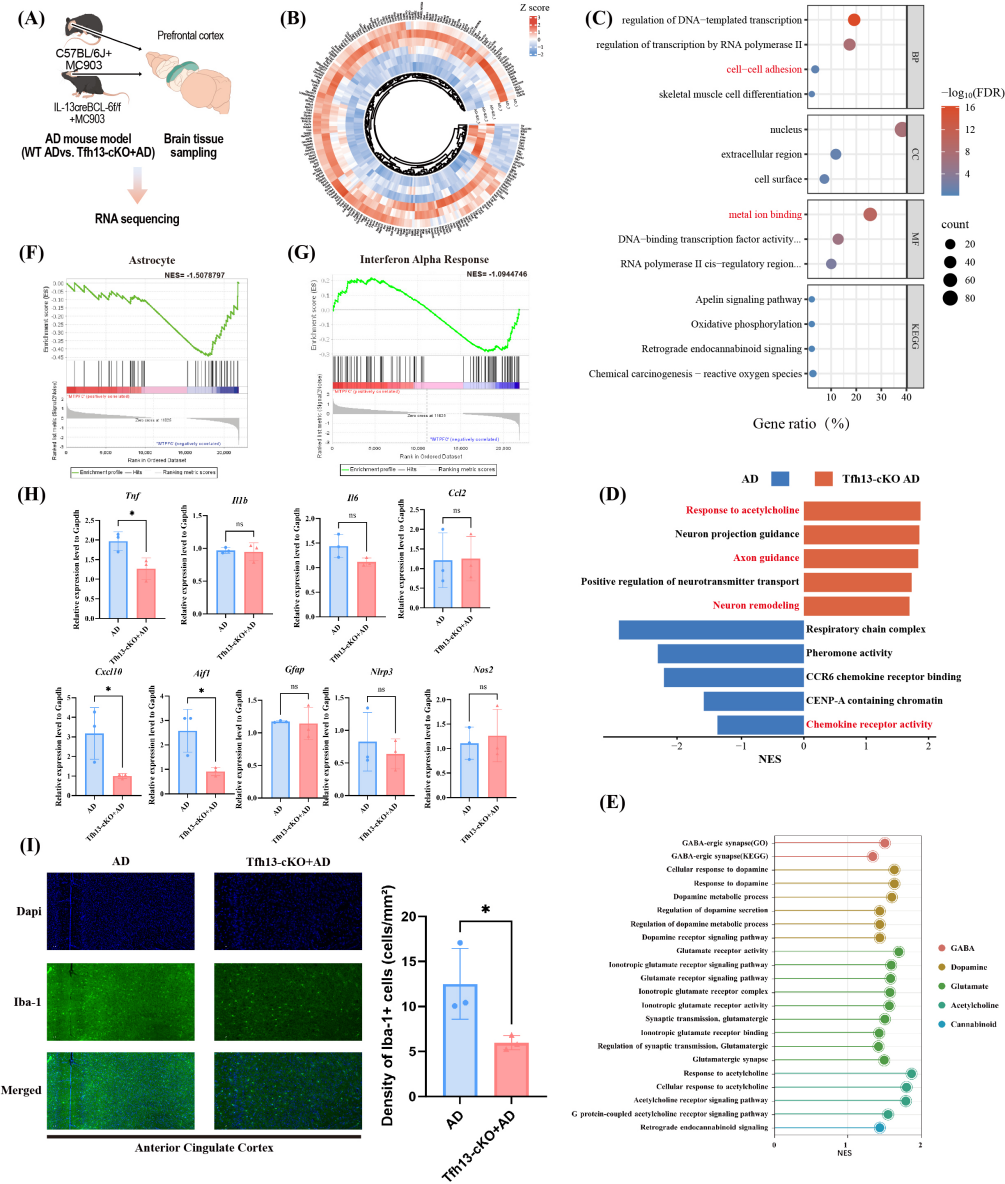
Peripheral inflammatory relief is associated with a marked suppression of neuroinflammatory signaling and increased synaptic plasticity in the prefrontal cortex of AD mice. **(A)** Brain tissue sampling of the prefrontal cortex from AD and Tfh13-cKO AD mice. **(B)** Circular heatmap showing the expression profiles of DEGs. Each spoke represents an individual gene, and each concentric ring represents a sample from either the AD group or the Tfh13-cKO AD group. Gene expression levels are visualized as Z-scores calculated by row-scaling the normalized counts. The color scale indicates the relative expression level, where red (positive Z-score) represents up-regulation and blue (negative Z-score) represents down-regulation relative to the mean. **(C)** Functional analysis of DEGs between mice of AD group and those of Tfh13-cKO AD group. **(D)** GSEA-GO analysis of expression matrix from prefrontal cortex of AD group and Tfh13-cKO AD group. GO terms upregulated in each group were displayed. **(E)** Enriched neurotransmitter pathways in GSEA pathway enrichment analysis. All these pathways showed significant upregulation in PFC of Tfh13-cKO AD mice. **(F)** Cell type enrichment analysis of expression matrix from PFC of AD mice and Tfh13-cKO AD mice. Astrocyte is the only cell type significantly downregulated in PFC of Tfh13-cKO AD mice. **(G)** GSEA-Hallmark pathway analysis reveals downregulation of interferon alpha response pathway in PFC of Tfh13-cKO AD mice. **(H)** RT-qPCR validation of neuroinflammatory and glial activation markers in the PFC of AD and Tfh13-cKO mice. **(I)** Representative images of DAPI (blue, nuclei) and Iba-1 (green, microglia) staining in the ACC of AD and Tfh13-cKO AD mice (left, scale bar = 100 μm). The bar graph (right) shows the density of Iba-1+ microglia (cells/mm^2^) in the ACC. A significant decrease in microglial density was observed in the Tfh13-cKO AD group compared to the WT-AD group. Data are presented as mean ± SEM (n = 3 per group). Statistical significance was determined using an unpaired two-tailed Student’s t-test; P < 0.05 was considered significant. *P < 0.05, **P < 0.01; ns, not significant.

To investigate whether alterations in peripheral inflammatory status impact central neuroinflammation in AD, we collected prefrontal cortex (PFC) samples from wild-type AD mice and Tfh13-cKO AD mice for RNA-seq analysis (see **Figure 6A**). Differential gene expression analysis revealed that 19 genes were upregulated and 228 genes were downregulated in Tfh13-cKO AD mice (see **Figure 6B**). Functional enrichment analysis indicated that these differentially expressed genes were primarily associated with cell−cell adhesion (see **Figure 6C**). Within the context of the central nervous system, the enrichment of these pathways may reflect changes in the immunological state and synaptic connectivity of the PFC in Tfh13-cKO AD mice. Further GSEA-GO analysis showed that pathways related to synaptic plasticity, such as axon guidance and neuron remodeling, were upregulated in the PFC of Tfh13-cKO AD mice, whereas inflammation-associated pathways, including chemokine receptor signaling, were downregulated (see **Figure 6D**). In addition, multiple neurotransmitter system-related pathways were significantly upregulated in the PFC of Tfh13-cKO AD mice, suggesting that attenuation of peripheral inflammation enhances synaptic plasticity in AD mice (see **Figure 6E**). Interestingly, we also observed downregulation of both astrocyte-related gene sets (see **Figure 6F**) and the “interferon-alpha response” pathway (see **Figure 6G**) in the prefrontal cortex of Tfh13-cKO AD mice, which exhibit attenuated peripheral inflammation. Further validation using RT-qPCR showed that *Tnf* along with *Cxcl10* were significantly downregulated in Tfh13-cKO AD mice, while *Il6* exhibited a downward trend. The mRNA expression of the microglial marker *Aif1* was also significantly decreased, indicating attenuated microglial activation levels (see **Figure 6H**). We next performed immunofluorescence staining on brain sections from Tfh13-cKO AD mice and conducted a quantitative analysis of Iba-1+ microglia in the target region. The results demonstrated that, compared to the AD group, the density of Iba-1+ microglia within ACC of Tfh13-cKO AD mice was significantly reduced (see **Figure 6I**). This histological evidence is highly consistent with downregulation of Aif1 mRNA levels, collectively confirming that the attenuation of peripheral allergic inflammation is accompanied by a concomitant decrease in central neuroinflammation in AD mice.”

## 4 Discussion

By integrating cutting-edge methodologies, including neuroimaging transcriptomics and experimental validation in mouse models, this study elucidated the neuroinflammatory mechanisms underlying aberrant activation patterns in the central nervous system of AD patients. Our findings demonstrate that neuroinflammatory processes mediated by astrocytes and microglia, together with dysfunctions in dopaminergic and GABAergic systems, play pivotal roles in the abnormal central nervous system activation observed in AD patients.

Through cell type–specific analyses, we observed that, compared with healthy controls, genes associated with astrocytes were significantly upregulated in LSFG of AD patients. In parallel, genes related to activation of microglia and endothelial cells were also markedly upregulated in sensorimotor regions exhibiting abnormal functional connectivity with the LSFG. Astrocytes and microglia are key cellular mediators of neuroinflammation, exerting their effects through the secretion of cytokines and inflammatory mediators (32). During neuroinflammatory processes, bidirectional communication between astrocytes and endothelial cells can increase blood–brain barrier permeability, thereby facilitating the infiltration of peripheral immune cells (33, 34). Accumulating evidence suggests that alterations in brain network function represent one of the most direct manifestations of exacerbated neuroinflammation (35, 36). For instance, microglial activation can shape neural networks via mechanisms such as synaptogenesis, synaptic pruning, and the release of neuromodulatory factors, ultimately influencing interregional functional connectivity (37). These observations are consistent with our previous findings showing that the LSFG in AD patients exhibits higher DC values and abnormal functional connectivity with multiple sensorimotor-related brain regions. Furthermore, the spatial correlation between these imaging abnormalities and the expression of specific interleukin-related genes, such as IL3RA, IL13RA1, IL17RD, and IL33, provides molecular insight into this brain-body interaction. We interpret this association as an indication that under the conditions of peripheral inflammation in AD, circulating inflammatory mediators may modulate the CNS immune microenvironment through mechanisms such as altered BBB permeability. Although members of the interleukin family are traditionally regarded as key mediators of peripheral inflammation, emerging evidence indicates that they also play important roles within the central nervous system, including the regulation of neuroinflammation and synaptic plasticity (28–31). Together, these results indicate that neuroinflammation within the LSFG, mediated by the interplay between astrocytes and microglia, ultimately contributes to the aberrant central nervous system activation patterns observed in AD. Furthermore, GSEA of neuroimaging transcriptomic data from the LSFG of AD patients revealed significant upregulation of pathways related to inflammatory responses and oxidative stress, including “inflammatory response”, “asthma” and “oxidative phosphorylation”, thereby providing additional support for this interpretation.

To determine whether glial cell activation–mediated neuroinflammation occurs in the context of atopic dermatitis, we employed an MC903-induced AD mouse model for mechanistic investigation. In the human brain, the left superior frontal gyrus (LSFG) is an important component of the prefrontal cortex. however, a direct LSFG homolog is not defined in the murine brain. Therefore, the prefrontal cortex was selected as the corresponding region of interest for subsequent analyses. Differentially expressed genes between AD and control mice were predominantly enriched in inflammation- and immune response–related pathways. Consistently, GSEA demonstrated a significant upregulation of interferon-related gene sets in the prefrontal cortex of AD mice compared with control mice, closely mirroring the neuroimaging transcriptomic findings observed in the LSFG of AD patients. Notably, in the animal model, we found that the expression of the astrocytic activation marker Gfap and the microglial activation marker Iba-1 was significantly elevated in the prefrontal cortex of AD mice relative to controls. In parallel, RT–qPCR analyses showed that classical neuroinflammatory markers, including Tnf-α, Il-6, and Cxcl10, were also markedly upregulated in the prefrontal cortex of AD mice. Furthermore, we selected ACC—a key subregion within PFC previously reported to be significantly activated in the brains of AD patients as our target region for immunofluorescence staining. Our analysis revealed a significant increase in the number of microglia in AD mice, which also exhibited a distinct activated phenotype. As discussed above, microglia are regarded as the principal effector cells in neuroinflammation, while proinflammatory cytokines such as Tnf-α, Il-6, and Cxcl10 may act as effector molecules released by activated microglia to amplify inflammatory responses (38–40). Astrocytes, in turn, may respond to these proinflammatory cues and modulate the functional states of neighboring cells, thereby contributing to the persistence of neuroinflammation (41). Collectively, these findings suggest that under the background of peripheral inflammation associated with atopic dermatitis, the murine prefrontal cortex likewise exhibits a neuroinflammatory phenotype characterized by glial activation. The absence of significant changes in Nlrp3 and Nos2 expression may indicate that the inflammatory response has not progressed to a stage dominated by inflammasome activation or iNOS-driven oxidative stress. Moreover, we observed a downregulation of synaptic plasticity–related pathways in the prefrontal cortex of AD mice. Given that loss of synaptic plasticity is widely regarded as a key concomitant feature and pathological consequence of neuroinflammation, this observation further supports the above interpretation.

However, the factors responsible for triggering neuroinflammation in the prefrontal cortex of AD patients and in AD mouse models remain largely unclear. Prevailing concepts in the field of brain–body interactions propose that, under conditions of peripheral inflammatory diseases, circulating inflammatory mediators may induce blood–brain barrier (BBB) disruption, thereby facilitating the development of central neuroinflammation (42, 43). Previous studies using systemic inflammation models have demonstrated that proinflammatory mediators, such as Il-1β, Tnf-α, and Il-6, can act on cerebrovascular endothelial cells to disturb the regulation of tight junctions, ultimately compromising BBB integrity. In turn, circulating inflammatory mediators that gain access to the central nervous system are thought to trigger secondary cytokine release and activate resident microglia and astrocytes, culminating in neuroinflammatory responses. This paradigm has been substantiated across multiple experimental models, including lipopolysaccharide (LPS)–induced systemic inflammation and dextran sodium sulfate (DSS)–induced chronic intestinal inflammation (44). Interestingly, Thomson et al. reported that two distinct Toll-like receptor (TLR)–dependent models in mice—namely LPS-induced systemic inflammation and imiquimod-induced dermatitis—both resulted in the upregulation of type I interferon–related pathways in the brain (17). Although this study did not explicitly characterize neuroinflammatory phenotypes in the mouse brain, it nonetheless suggests that cutaneous inflammation, similar to systemic peripheral inflammation, may exert a potential transcriptomic reprogramming effect on the central nervous system. Consistent with this notion, we observed a significant upregulation of type I interferon signaling in both the neuroimaging transcriptomic data from the left superior frontal gyrus of AD patients and the transcriptomic profiles of AD mouse models. Growing evidence suggests that Type-I interferon response play a dual role in the CNS, acting as essential homeostatic regulators at basal levels while driving neuroinflammation when oversecreted (45, 46). Notably, microglial cells and astrocytes are identified as the primary producers of Type-I interferon in the CNS (47). In a chronic stress model, Tripathi et al. demonstrated that elevated Type-I interferon signaling triggers microglial activation and subsequent synaptic loss in the PFC, leading to social behavioral deficits, which could be reversed by conditional deletion of the interferon receptor (IFNAR) in microglia (48, 49). This mechanism aligns with our observation of downregulated synaptic plasticity-related pathways in the PFC of AD mice, suggesting that the interferon-microglia axis may represent a crucial link between peripheral allergic inflammation and the impairment of central synaptic function. Specifically, we tentatively propose a cascading neuroinflammatory model whereby elevated peripheral inflammation in AD might trigger cerebrovascular endothelial activation or alter blood-brain barrier permeability, potentially inducing the local release of Type-I interferon within the CNS. The interferon may, in turn, act on microglia and astrocytes to facilitate aberrant synaptic pruning and further contribute to the maintenance of a chronic neuroinflammatory state in the prefrontal cortex. However, further experimental validation is required to confirm this hypothesis. Taken together, these findings imply that, under peripheral inflammatory conditions, type I interferon signaling may function as an initiating factor or an active participant in neuroinflammatory processes, rather than merely representing a downstream consequence of systemic TLR activation. Taken together, we propose that peripheral inflammation in AD patients and mouse models may drive the activation of central nervous system neuroinflammation, with interferon signaling pathways playing a critical role in the initiation and/or propagation of these responses.

To further investigate the link between neuroinflammation in the PFC and peripheral immune alterations in AD, we established a calcipotriol-induced AD model utilizing Tfh13-cKO mice. Uthaman Gowthaman et al. demonstrated, using a T-Dock8^-^/^-^ hyper-IgE mouse model, that Tfh13 cells—rather than other T follicular helper subsets—play a critical role in the generation of high-affinity IgE (20). Conditional ablation of Tfh13 cells abrogated high-affinity IgE production and markedly attenuated the severity of allergic inflammation, findings that have been consistently validated across multiple allergic disease models (50, 51). In line with these observations, compared with wild-type (WT) AD mice, Tfh13-cKO AD mice exhibited significantly reduced skin lesion severity. Moreover, effector CD4^+^ T cells and germinal center B cells in peripheral lymphoid organs, as well as circulating IgE levels in peripheral blood, showed a downward trend, indicating a substantially attenuated peripheral inflammatory state in Tfh13-cKO mice subjected to calcipotriol-induced AD modeling. Further comparative analyses of brain transcriptomic profiles and inflammation-related gene expression between Tfh13-cKO AD mice and WT AD mice revealed that the expression of proinflammatory mediators, including Tnf-α and Cxcl10, as well as the microglial activation marker Aif1, was significantly downregulated in the prefrontal cortex of Tfh13-cKO AD mice, with Il-6 expression also showing a decreasing trend. Meanwhile, quantitative immunofluorescence analysis revealed a significant reduction in microglial density within the ACC of Tfh13-cKO AD mice. Notably, synaptic plasticity–related pathways were significantly upregulated in Tfh13-cKO AD mice relative to WT AD mice, whereas type I interferon signaling exhibited a downward trend. Collectively, these findings suggest that microglia-mediated neuroinflammation is alleviated under conditions of reduced peripheral allergic inflammation in AD. Since Tfh13 cells are strictly restricted to secondary lymphoid organs and do not infiltrate the CNS, the attenuated neuroinflammation observed in Tfh13-cKO mice strongly supports the notion that peripheral immune status is linked to CNS pathology. Accordingly, we propose that chronic peripheral inflammation in AD patients may trigger neuroinflammatory processes within the LSFG, leading to impaired synaptic function and disrupted functional connectivity between the LSFG and other brain regions, ultimately giving rise to an aberrant LSFG-centered central nervous system activation pattern.

To further dissect the aberrant central nervous system activation patterns in AD patients from a neurotransmitter perspective, we performed an MRI–nuclear cross-modal correlation analysis using LSFG as the region of interest (ROI). Spatial correlation analysis revealed that neural correlates of the dopaminergic system were highly associated with neuroimaging parameter differences observed between AD patients and healthy controls. Dopamine and its metabolites have been extensively studied as emotion-related neurotransmitters. However, emerging evidence indicates that dopamine also serves as an important modulator of immune function (52). In the context of cutaneous immunity, dopamine receptor antagonists have been shown to suppress both immediate-type reactions (ITR) and late-phase reactions (LPR) in experimental models of atopic dermatitis (53). Clinical studies have further reported alterations in peripheral dopamine metabolic pathways in AD patients, with significantly elevated plasma dopamine levels compared with healthy controls that markedly decrease following anti-inflammatory treatment (54). In addition, reward-related neural circuits closely linked to dopaminergic signaling may contribute to the itch–scratch cycle in AD patients. This notion is consistent with our correlation analyses showing reduced expression of the dopamine transporter (DAT) in the LSFG. DAT is selectively expressed in dopaminergic neurons and regulates dopamine reuptake from the synaptic cleft into presynaptic terminals; thus, decreased DAT expression may indicate enhanced dopaminergic signaling within the LSFG, potentially facilitating scratching behavior (55, 56). The increased expression of dopamine D2 receptors in brain regions exhibiting enhanced degree-centrality–based functional connectivity further supports the presence of augmented dopaminergic signaling. 18F-DOPA, a L-DOPA analog with similar metabolic characteristics, serves as an *in vivo* indicator of dopamine synthesis capacity (57). Notably, we observed reduced FDOPA levels in the LSFG, suggesting that inflammation-associated pruritus–induced elevations in dopamine signaling may exert a negative feedback effect on dopamine synthesis in this region. Collectively, these findings imply that chronic pruritus driven by peripheral inflammation may enhance dopaminergic neurotransmission predominantly through inhibition of dopamine reuptake rather than increased dopamine synthesis, thereby promoting scratching behavior. The precise role of dopamine in the neuroimmune mechanisms of AD warrants further investigation. Of note, the non-selective DAT inhibitor bupropion has shown beneficial effects in the treatment of AD (58). In non-depressed AD patients, bupropion administration was associated with a reduction in lesional body surface area, whereas discontinuation of the drug led to an increase in lesion extent relative to baseline (59). Bupropion may normalize underlying neuroendocrine, immune, or catecholaminergic dysregulation in AD, which could represent a potential mechanism underlying its therapeutic effects on AD skin lesions.

In addition, increasing attention has been directed toward the role of the γ-aminobutyric acid (GABA)ergic system in neuroinflammation, although the underlying mechanisms remain controversial. Under conditions of chronic inflammation, the GABAergic neurotransmitter system may engage in complex bidirectional interactions with neuroinflammatory processes. A review by Crowley et al. summarized that although GABAergic currents are generally reduced in response to inflammatory signaling, the effects of inflammation on GABA_A_ ​receptor expression appear to be heterogeneous (60). For example, TNF-α upregulation associated with chronic pain has been shown to increase GABA_A_ receptor expression. Similarly, lipopolysaccharide (LPS)-induced acute neuroinflammation leads to the upregulation of GABAergic pathways, including GABA_A_ receptors, and subsequent treatment with GABA receptor agonists attenuates excessive microglial activation, suggesting a potential anti-inflammatory role of GABAergic signaling in neuroinflammation (61). In the present study, increased expression of GABA_A_ receptors was observed in brain regions exhibiting abnormal functional connectivity with the LSFG in AD patients. This alteration may likewise reflect a compensatory or protective anti-inflammatory response of the GABAergic system under neuroinflammatory conditions.

This study has several limitations. First, the cross-sectional design of the neuroimaging analyses limits causal inference, and the relatively small sample size may affect the generalizability of the findings. Future studies with larger, more diverse cohorts and longitudinal designs are warranted.

Overall, this study underscores the importance of peripheral–central immune interactions in the pathogenesis of atopic dermatitis and provides an integrative framework that may inform more precise and individualized therapeutic strategies.

## Data Availability

The datasets presented in this study can be found in online repositories. The names of the repository/repositories and accession number(s) can be found below: https://dataview.ncbi.nlm.nih.gov/object/PRJNA1469276?reviewer=jf6g0ho4r8t0o49s70i5vq32b0.

## References

[1] Bieber T . Atopic dermatitis: an expanding therapeutic pipeline for a complex disease. Nat Rev Drug Discov. (2022) 21:21–40. doi: 10.1038/s41573-021-00266-6 34417579 PMC8377708

[2] Langan SM Irvine AD Weidinger S . Atopic dermatitis. Lancet (London England). (2020) 396:345–60. doi: 10.1016/s0140-6736(20)31286-1 32738956

[3] Oetjen LK Mack MR Feng J Whelan TM Niu H Guo CJ . Sensory neurons co-opt classical immune signaling pathways to mediate chronic itch. Cell. (2017) 171:217–28 e13. doi: 10.4049/jimmunol.198.supp.63.3 28890086 PMC5658016

[4] Wilson SR The L Batia LM Beattie K Katibah GE McClain SP . The epithelial cell-derived atopic dermatitis cytokine TSLP activates neurons to induce itch. Cell. (2013) 155:285–95. doi: 10.1016/j.cell.2013.08.057 24094650 PMC4041105

[5] Granstein RD Wagner JA Stohl LL Ding W . Calcitonin gene-related peptide: key regulator of cutaneous immunity. Acta Physiol (Oxf). (2015) 213:586–94. doi: 10.1111/apha.12442 25534428 PMC4308419

[6] Steinhoff M Ahmad F Pandey A Datsi A AlHammadi A Al-Khawaga S . Neuroimmune communication regulating pruritus in atopic dermatitis. J Allergy Clin Immunol. (2022) 149:1875–98. doi: 10.1016/j.jaci.2022.03.010 35337846

[7] Raap M Rudrich U Stander S Gehring M Kapp A Raap U . Substance P activates human eosinophils. Exp Dermatol. (2015) 24:557–9. doi: 10.1111/exd.12717 25865137

[8] Ding W Manni M Stohl LL Zhou XK Wagner JA Granstein RD . Pituitary adenylate cyclase-activating peptide and vasoactive intestinal polypeptide bias Langerhans cell Ag presentation toward Th17 cells. Eur J Immunol. (2012) 42:901–11. doi: 10.1002/eji.201141958 22531916 PMC3615422

[9] Shiratori-Hayashi M Koga K Tozaki-Saitoh H Kohro Y Toyonaga H Yamaguchi C . STAT3-dependent reactive astrogliosis in the spinal dorsal horn underlies chronic itch. Nat Med. (2015) 21:927–31. doi: 10.1038/nm.3912 26193341

[10] Liu T Han Q Chen G Huang Y Zhao L-X Berta T . Toll-like receptor 4 contributes to chronic itch, alloknesis, and spinal astrocyte activation in male mice. Pain. (2016) 157:806–17. doi: 10.1097/j.pain.0000000000000439 26645545 PMC4946956

[11] Schneider G Ständer S Burgmer M Driesch G Heuft G Weckesser M . Significant differences in central imaging of histamine-induced itch between atopic dermatitis and healthy subjects. Eur J Pain (London England). (2008) 12:834–41. doi: 10.1201/9780367854706-25 18203636

[12] Ishiuji Y Coghill RC Patel TS Oshiro Y Kraft RA Yosipovitch G . Distinct patterns of brain activity evoked by histamine-induced itch reveal an association with itch intensity and disease severity in atopic dermatitis. Br J Dermatol. (2009) 161:1072–80. doi: 10.1111/j.1365-2133.2009.09308.x 19663870 PMC2784001

[13] Wu H Dai W Hong Z Qin Y Yang M Wang B . Higher-order sensorimotor circuit of the whole-brain functional network involved in pruritus regulation in atopic dermatitis. J Eur Acad Dermatol Venereology: JEADV. (2024) 38:873–82. doi: 10.1111/jdv.19691 38069553

[14] Li C-Y Chang W-C Chen M-H Tu P-C Chen T-L Chen C-C . Correlation of disease severity, proinflammatory cytokines, and reduced brain gray matter volumes in patients with atopic dermatitis. Dermatitis: Contact Atopic Occupational Drug. (2024) 35:489–97. doi: 10.1089/derm.2023.0340 38634841

[15] Sun Y Koyama Y Shimada S . Inflammation from peripheral organs to the brain: how does systemic inflammation cause neuroinflammation? Front Aging Neurosci. (2022) 14:903455. doi: 10.3389/fnagi.2022.903455 35783147 PMC9244793

[16] Hoogland IC Houbolt C van Westerloo DJ van Gool WA van de Beek D . Systemic inflammation and microglial activation: systematic review of animal experiments. J Neuroinflamm. (2015) 12:114. doi: 10.1186/s12974-015-0332-6 26048578 PMC4470063

[17] Thomson CA McColl A Cavanagh J Graham GJ . Peripheral inflammation is associated with remote global gene expression changes in the brain. J Neuroinflamm. (2014) 11:73. doi: 10.1186/1742-2094-11-73 24708794 PMC4022192

[18] Arnatkeviciute A Markello RD Fulcher BD Misic B Fornito A . Toward best practices for imaging transcriptomics of the human brain. Biol Psychiatry. (2023) 93:391–404. doi: 10.1016/j.biopsych.2022.10.016 36725139

[19] Fornito A Arnatkevičiūtė A Fulcher BD . Bridging the gap between connectome and transcriptome. Trends Cognit Sci. (2019) 23:34–50. doi: 10.31219/osf.io/fj5tg 30455082

[20] Gowthaman U Chen JS Zhang B Flynn WF Lu Y Song W . Identification of a T follicular helper cell subset that drives anaphylactic IgE. Science. (2019) 365:eaaw6433. doi: 10.1126/science.aaw6433 31371561 PMC6901029

[21] Kulthanan K Tuchinda P Nitiyarom R Chunharas A Chantaphakul H Aunhachoke K . Clinical practice guidelines for the diagnosis and management of atopic dermatitis. Asian Pacific J Allergy Immunol. (2021) 39:145–55. doi: 10.12932/AP-010221-1050 34246205

[22] Desikan RS Ségonne F Fischl B Quinn BT Dickerson BC Blacker D . An automated labeling system for subdividing the human cerebral cortex on MRI scans into gyral based regions of interest. NeuroImage. (2006) 31:968–80. doi: 10.1016/j.neuroimage.2006.01.021 16530430

[23] Martins D Giacomel A Williams SCR Turkheimer F Dipasquale O Veronese M . Imaging transcriptomics: convergent cellular, transcriptomic, and molecular neuroimaging signatures in the healthy adult human brain. Cell Rep. (2021) 37:110173. doi: 10.1016/j.celrep.2021.110173 34965413

[24] Dukart J Holiga S Rullmann M Lanzenberger R Hawkins PCT Mehta MA . JuSpace: a tool for spatial correlation analyses of magnetic resonance imaging data with nuclear imaging derived neurotransmitter maps. Hum Brain Mapp. (2021) 42:555–66. doi: 10.1101/2020.04.17.046300 33079453 PMC7814756

[25] Love MI Huber W Anders S . Moderated estimation of fold change and dispersion for RNA-seq data with DESeq2. Genome Biol. (2014) 15:550. doi: 10.1186/s13059-014-0550-8 25516281 PMC4302049

[26] Sherman BT Hao M Qiu J Jiao X Baseler MW Lane HC . DAVID: a web server for functional enrichment analysis and functional annotation of gene lists (2021 update). Nucleic Acids Res. (2022) 50:W216–W21. doi: 10.1093/nar/gkac194 35325185 PMC9252805

[27] Huang DW Sherman BT Lempicki RA . Systematic and integrative analysis of large gene lists using DAVID bioinformatics resources. Nat Protoc. (2009) 4:44–57. doi: 10.1038/nprot.2008.211 19131956

[28] Rao X Hua F Zhang L Lin Y Fang P Chen S . Dual roles of interleukin-33 in cognitive function by regulating central nervous system inflammation. J Transl Med. (2022) 20:369. doi: 10.1186/s12967-022-03570-w 35974336 PMC9382782

[29] Singh Gautam A Kumar Singh R . Therapeutic potential of targeting IL-17 and its receptor signaling in neuroinflammation. Drug Discov Today. (2023) 28:103517. doi: 10.1016/j.drudis.2023.103517 36736763

[30] Khan AW Farooq M Hwang MJ Haseeb M Choi S . Autoimmune neuroinflammatory diseases: role of interleukins. Int J Mol Sci. (2023) 24:7960. doi: 10.3390/ijms24097960 37175665 PMC10178921

[31] Milovanovic J Arsenijevic A Stojanovic B Kanjevac T Arsenijevic D Radosavljevic G . Interleukin-17 in chronic inflammatory neurological diseases. Front Immunol. (2020) 11:947. doi: 10.3389/fimmu.2020.00947 32582147 PMC7283538

[32] Linnerbauer M Wheeler MA Quintana FJ . Astrocyte crosstalk in CNS inflammation. Neuron. (2020) 108:608–22. doi: 10.1016/j.neuron.2020.08.012 32898475 PMC7704785

[33] Liu LR Liu JC Bao JS Bai QQ Wang GQ . Interaction of microglia and astrocytes in the neurovascular unit. Front Immunol. (2020) 11:1024. doi: 10.3389/fimmu.2020.01024 32733433 PMC7362712

[34] Abbott NJ Rönnbäck L Hansson E . Astrocyte-endothelial interactions at the blood-brain barrier. Nat Rev Neurosci. (2006) 7:41–53. doi: 10.1038/nrn1824 16371949

[35] Goldsmith DR Bekhbat M Mehta ND Felger JC . Inflammation-related functional and structural dysconnectivity as a pathway to psychopathology. Biol Psychiatry. (2023) 93:405–18. doi: 10.1016/j.biopsych.2022.11.003 36725140 PMC9895884

[36] Kraynak TE Marsland AL Wager TD Gianaros PJ . Functional neuroanatomy of peripheral inflammatory physiology: a meta-analysis of human neuroimaging studies. Neurosci Biobehav Rev. (2018) 94:76–92. doi: 10.1016/j.neubiorev.2018.07.013 30067939 PMC6363360

[37] Salter MW Beggs S . Sublime microglia: expanding roles for the guardians of the CNS. Cell. (2014) 158:15–24. doi: 10.1016/j.cell.2014.06.008 24995975

[38] Del Moro L Brunetta E Gershwin ME Selmi C . Microglia and myeloperoxidase in neuroinflammatory and neurodegenerative diseases. Curr Opin Immunol. (2025) 97:102660. doi: 10.1016/j.coi.2025.102660 40972344

[39] Shafi A Akmal M Sethi A Chauhdary Z . A mechanistic insight of neuro-inflammation signaling pathways and implication in neurodegenerative disorders. Inflammopharmacology. (2025) 34:309–18. doi: 10.1007/s10787-025-02041-0 41266692

[40] Bufi AA Di Stefano J Papait A Silini AR Parolini O Ponsaerts P . The central role of CXCL10-CXCR3 signaling in neuroinflammation and neuropathology. Cytokine Growth Factor Rev. (2025) 84:20–34. doi: 10.1016/j.cytogfr.2025.05.003 40473519

[41] Rothhammer V Quintana FJ . Control of autoimmune CNS inflammation by astrocytes. Semin Immunopathol. (2015) 37:625–38. doi: 10.1007/s00281-015-0515-3 26223505 PMC4618768

[42] Abbott NJ Patabendige AA Dolman DE Yusof SR Begley DJ . Structure and function of the blood-brain barrier. Neurobiol Dis. (2010) 37:13–25. doi: 10.1016/j.nbd.2009.07.030 19664713

[43] Hurtado-Alvarado G Dominguez-Salazar E Pavon L Velazquez-Moctezuma J Gomez-Gonzalez B . Blood-brain barrier disruption induced by chronic sleep loss: low-grade inflammation may be the link. J Immunol Res. (2016) 2016:4576012. doi: 10.1155/2016/4576012 27738642 PMC5050358

[44] Zonis S Pechnick RN Ljubimov VA Mahgerefteh M Wawrowsky K Michelsen KS . Chronic intestinal inflammation alters hippocampal neurogenesis. J Neuroinflamm. (2015) 12:65. doi: 10.1186/s12974-015-0281-0 25889852 PMC4403851

[45] McGlasson S Jury A Jackson A Hunt D . Type I interferon dysregulation and neurological disease. Nat Rev Neurol. (2015) 11:515–23. doi: 10.1038/nrneurol.2015.143 26303851

[46] Roy ER Wang B Wan YW Chiu G Cole A Yin Z . Type I interferon response drives neuroinflammation and synapse loss in Alzheimer disease. J Clin Invest. (2020) 130:1912–30. doi: 10.1172/jci133737 31917687 PMC7108898

[47] Viengkhou B White MY Cordwell SJ Campbell IL Hofer MJ . A novel phosphoproteomic landscape evoked in response to type I interferon in the brain and in glial cells. J Neuroinflamm. (2021) 18:237. doi: 10.1186/s12974-021-02277-x 34656141 PMC8520650

[48] Tripathi A Whitehead C Surrao K Pillai A Madeshiya A Li Y . Type 1 interferon mediates chronic stress-induced neuroinflammation and behavioral deficits via complement component 3-dependent pathway. Mol Psychiatry. (2021) 26:3043–59. doi: 10.1038/s41380-021-01065-6 33833372 PMC8497654

[49] Tripathi A Bartosh A Mata J Jacks C Madeshiya AK Hussein U . Microglial type I interferon signaling mediates chronic stress-induced synapse loss and social behavior deficits. Mol Psychiatry. (2025) 30:423–34. doi: 10.1038/s41380-024-02675-6 39095477

[50] Yu B Pei C Peng W Zheng Y Fu Y Wang X . Microbiota-derived butyrate alleviates asthma via inhibiting Tfh13-mediated IgE production. Signal Transduct Target Ther. (2025) 10:181. doi: 10.1038/s41392-025-02263-2 40473603 PMC12141656

[51] Chandrakar P Nelson CS Podesta MA Cavazzoni CB Gempler M Lee JM . Progressively differentiated T(FH)13 cells are stabilized by JunB to mediate allergen germinal center responses. Nat Immunol. (2025) 26:473–83. doi: 10.1038/s41590-025-02077-y 39891019 PMC12169414

[52] Matt SM Gaskill PJ . Where is dopamine and how do immune cells see it?: Dopamine-mediated immune cell function in health and disease. J Neuroimmune Pharmacology: Off J Soc Neuroimmune Pharmacol. (2020) 15:114–64. doi: 10.1007/s11481-019-09851-4 31077015 PMC6842680

[53] Mori T Kabashima K Fukamachi S Kuroda E Sakabe J Kobayashi M . D1-like dopamine receptors antagonist inhibits cutaneous immune reactions mediated by Th2 and mast cells. J Dermatol Sci. (2013) 71:37–44. doi: 10.1016/j.jdermsci.2013.03.008 23639699

[54] Ishiuji Y . Addiction and the itch-scratch cycle. What do they have in common? Exp Dermatol. (2019) 28:1448–54. doi: 10.1111/exd.14029 31482585

[55] Setsu T Hamada Y Oikawa D Mori T Ishiuji Y Sato D . Direct evidence that the brain reward system is involved in the control of scratching behaviors induced by acute and chronic itch. Biochem Biophys Res Commun. (2021) 534:624–31. doi: 10.1016/j.bbrc.2020.11.030 33220930

[56] Yuan L Liang TY Deng J Sun YG . Dynamics and functional role of dopaminergic neurons in the ventral tegmental area during itch processing. J Neuroscience: Off J Soc For Neurosci. (2018) 38:9856–69. doi: 10.1523/jneurosci.1483-18.2018 30266741 PMC6596242

[57] Snow BJ Tooyama I McGeer EG Yamada T Calne DB Takahashi H . Human positron emission tomographic [18F]fluorodopa studies correlate with dopamine cell counts and levels. Ann Neurol. (1993) 34:324–30. doi: 10.1002/ana.410340304 8363349

[58] Eskeland S Halvorsen JA Tanum L . Antidepressants have anti-inflammatory effects that may be relevant to dermatology: A systematic review. Acta Dermato-Venereologica. (2017) 97:897–905. doi: 10.2340/00015555-2702 28512664

[59] Modell JG Boyce S Taylor E Katholi C . Treatment of atopic dermatitis and psoriasis vulgaris with bupropion-SR: A pilot study. Psychosomatic Med. (2002) 64:835–40. doi: 10.1097/01.psy.0000021954.59258.9b 12271115

[60] Crowley T Cryan JF Downer EJ O'Leary OF . Inhibiting neuroinflammation: The role and therapeutic potential of GABA in neuro-immune interactions. Brain Behavior Immun. (2016) 54:260–77. doi: 10.1016/j.bbi.2016.02.001 26851553

[61] Jiang J Tang B Wang L Huo Q Tan S Misrani A . Systemic LPS-induced microglial activation results in increased GABAergic tone: A mechanism of protection against neuroinflammation in the medial prefrontal cortex in mice. Brain Behavior Immun. (2022) 99:53–69. doi: 10.1016/j.bbi.2021.09.017 34582995

