# Supplementary material for: Cross-species transcriptomic evidence for peripheral–central immune crosstalk in atopic dermatitis
Source: Front Immunol. 2026 Jun 26;17:1790710. doi: 10.3389/fimmu.2026.1790710 (PMC13349758; doi:10.3389/fimmu.2026.1790710)
Supplement: Supplementary file 1 [file DataSheet1.docx]

Supplementary Material

# Supplementary Data

**Supplementary methods**

**Supplementary method 1: Eligibility criteria and exclusion details for subject selection**

Participants were recruited based on the following eligibility criteria: (1) confirmed diagnosis of AD in accordance with the international criteria of Hanifin and Rajka (1), with a duration of at least 1 year; (2) Severity Scoring of Atopic Dermatitis (SCORAD) score above 25(2); (3) males or females aged between 18 and 60 years; (4) Han nationality; (5) able to comprehend the instructions of clinical dermatologists; and (6) right-handed, according to the Edinburgh Handedness Inventory(3). The exclusion criteria included: (1) presence of neurodegenerative or psychiatric disorders; (2) co-existing skin disease; (3) severe somatic disorders, including diabetes and so on; (4) any contraindication to magnetic resonance image (MRI); (5) head motion >2 mm in MRI scans and (6) pregnant or breastfeeding females.

**Supplementary method 2: Psychophysical Measurements**

Prior to imaging, AD participants were evaluated in person by a study physician and provided informed consent. Severity Scoring of Atopic Dermatitis (SCORAD) was used to assess symptoms of AD(4). Subjective symptoms (average pruritus during the past 3 days) were scored by AD participants on a visual analogue scale (VAS) in SCORAD from 0 (no pruritus) to 10 (the most severe imaginable pruritus). Pruritus VAS scored higher than or equal to 7 points was described as severe pruritus(5).

The diagnosis of major depressive disorder (MDD) or depressive disorder fulfilled the criteria for office coding in the Patient Health Questionnaire-9 (PHQ-9) (6). As the co-occurrence of depression could have a significant impact on fMRI data, we excluded 5 AD patients with comorbid MDD or depressive disorder according to PHQ-9.

**Supplementary method 3: Magnetic resonance image (MRI) scanning**

The participants were scanned using 3.0 T MRI equipment (MAGNETOM Skyra, Siemens Healthcare, Germany) with a standard 20-channel combination head and neck coil. Foam pads were employed to minimize the participants' head motion, and headphones were utilized to reduce noise during scanning. During the rs-fMRI scan, participants were instructed to keep their eyes closed, relax, and remain awake during scanning. To exclude organic brain lesions, we used simple T2-weighted imaging MRI. T1-weighted high-resolution structural images were obtained before the functional run using the three-dimensional fast spoiled gradient-echo sequence, with the following parameters: TR = 2530 ms, TE = 3.45 ms, TI = 1100 ms, FA= 7°, matrix size = 256 × 256, FOV = 256 mm × 256 mm, slice thickness = 1 mm, no gap and 192 sagittal slices. Functional images were acquired with the subsequent parameters: TR = 2000 ms, TE = 30 ms, FA = 90°, matrix size =64×64, FOV = 220 mm × 220 mm, slice thickness = 4.0 mm, 33 transverse slices, and 240 volumes. Rs- fMRI acquisitions were used to estimate head motion by applying the rigid-body transformation, and any participants who moved >2 mm were not included for further analysis. Four AD participants were excluded from the study due to this reason.

**Supplementary method 4: MRI Data processing**

Image preprocessing was performed by the Data Processing & Analysis of Brain Imaging toolbox (DPABI, V4.2, http://rfmri.org/dpabi) running on MATLAB R2021b software(7). The first 10 scanning volumes were removed to stabilize the magnetic resonance signal and reduce the impact of participants who did not adapt to the scanning environment. Slice timing and head motion were corrected for each participant, and those whose head motion exceeded 2 mm or head rotation exceeded 2° were excluded from further analysis. Next, the images were spatially normalized to the standard Montreal Neurological Institute template using warping parameters estimated from T1 images with a resampling standard voxel size of 3 mm × 3 mm × 3 mm. We used a 6 mm full-width at half-maximum Gaussian kernel to spatially smooth images and performed linear detrending. The nuisance signals, which included head motion effects (Friston 24-parameter model(8)), white matter, and cerebrospinal fluid, were regressed. Finally, band-pass filtering with a range of 0.01–0.08 Hz was applied to the time series of each voxel to remove the effects of very low-frequency drifts and high-frequency noise. The time series of each voxel for each participant was then transformed into the frequency domain using the fast Fourier transformation method to obtain the power spectrum.

Subsequently, we performed DC analysis using DPABI, which is a technique based on graph theory. DC has been extensively applied to investigate node characteristics of brain intrinsic connectivity networks in diverse pathologies(9).

**Supplementary method 5: Degree centrality analysis**

Voxel-wise degree centrality analysis was performed using the Data Processing & Analysis of Brain Imaging toolbox (DPABI, V4.2, http://rfmri.org/dpabi) running on MATLAB R2021b software(7). In the current analysis, each functional connection (Pearson correlation at r > 0.25) between voxels constitutes an edge. Degree centrality counts the number of edges connecting to a voxel. The second-level two- sample t-test was conducted with age, sex, education, and mean frame displacement (FD) Jenkinson as covariates. Multiple comparisons were corrected using the Guassian-Random-Field-Correction (GRF; voxel-level p<0.01, cluster-level p<0.05, two-tailed) with brainmask_05_61*73*61 in DPARSF(7). The coordinates of cluster centers were used to make regions of interest (ROIs) (sphere with r = 3mm). Then mean degree centrality values within these ROIs were computed for each AD participant.

**Supplementary method 6: Functional connectivity analysis**

ROI-based functional connectivity is a method used to investigate the functional connectivity between specific brain ROIs. In this study, ROIs were selected based on the degree centrality analysis results. The mean time series of each ROI was calculated, and the Pearson correlation coefficient was computed for each pair of regions to evaluate the strength of functional connectivity between them. To ensure the accuracy of the results, the correlation r-values were transformed into normally distributed Z-values using Fisher's Z transformation.

The mean time series of each ROI based on the degree centrality analysis results was calculated, and the Pearson correlation coefficient was computed for each pair of regions to evaluate the strength of functional connectivity between them in ROI-based functional connectivity. The Z-value maps of FC were then compared between AD participants group and the HCs using the second-level two-sample t-test while accounting for covariates including age, gender, education, and head motion parameters, with masks created in the one-sample t-test. Multiple comparisons were corrected using the false discovery rate (FDR) correction (p<0.05). The 3-mm radius spheres centered on the maximal peak voxel of brain regions displaying group differences in FC or DC were defined as ROIs, and their corresponding masks were created to extract the mean FC or DC values for subsequent analysis. Spearman correlation analysis was conducted between these fMRI data and both psychophysical measures and clinical symptoms. The significance was set at p < 0.05.

**Supplementary method 7: Brain gene expression data processing**

All of the regional genetic expression data came from the AHBA dataset (http://human.brain-map.org). Brain-wide gene expressions were measured in six postmortem brains, and totally 3702 samples were collected. Using the Imaging Transcriptomics toolbox (10, 11), based on the Abagen tool(12-14), we map the genetics expression data onto each brain regions. In brief, the gene expression data were processed as follows: 1) verifying the probe to gene annotations; 2) filtering the probes based on the intensity; 3) probe selection; 4) samples assignment to the Desikan-Killiany (DK) parcellation atlas(15) within 2mm Euclidean distance of a parcel; 5) scaled the regional expression value by using scaled robust sigmoid. Because the AHBA dataset included only two right hemisphere data, we only analyzed the data from left hemisphere. Afterwards, total 15633 distinct probes were survived and assigned to each brain parcellation. Thus, a mean of all samples in a region was calculated to obtain the gene expression matrix (83 regions x 15633 gene expression levels). Due to only two samples in the AHBA have included right hemisphere probes, we only analyzed the left hemisphere data in our current study.

**Supplementary method 8: Correlation with neurotransmitters**

We adopted JuSpace (https://github.com/juryxy/JuSpace) to investigate the spatial correlations of the t map with nuclear imaging derived measures covering various neurotransmitter systems including dopamine, serotonin, glutamate, GABA, acetylcholine, opioid, cannabinoid, noradrenaline, and fluorodopa (see Table S4 in the Supplementary materials) (16). Specifically, Pearson correlation coefficients between the t map and these neurotransmitter maps were calculated across 83 cerebral cortical regions derived from Neuromorphometrics atlas while adjusting for spatial autocorrelation and partial volume with the gray matter probability map. Exact P values were computed using spatial permutation-based null maps with 5000 permutations. Correlations were considered significant at P < 0.05.

**Supplementary method 9: Brain tissue sampling and RNA sequencing**

After all the mice were sacrificed, the brain tissues were harvested, and the prefrontal cortex were then isolated. All the tissues were freshly frozen and stored at -80°C for subsequent RNA Extraction. Total RNA was extracted using Trizol reagent kit (Invitrogen, Carlsbad, CA, USA) according to the manufacturer’s protocol. RNA quality was assessed on an Agilent 2100 Bioanalyzer (Agilent Technologies, Palo Alto, CA, USA) and checked using RNase free agarose gel electrophoresis. After total RNA was extracted, eukaryotic mRNA was enriched by Oligo(dT) beads. Then the enriched mRNA was fragmented into short fragments using fragmentation buffer and reversely transcribed into cDNA by using NEB Next Ultra RNA Library Prep Kit for Illumina (NEB#7530, New England Biolabs, Ipswich, MA, USA). The purified double-stranded cDNA fragments were end repaired, A base added, and ligated to Illumina sequencing adapters. The ligation reaction was purified with the AMPure XP Beads (1.0X). And polymerase chain reaction (PCR) amplified. The resulting cDNA library was sequenced using Illumina Novaseq6000 by Gene Denovo Biotechnology Co. (Guangzhou, China).

**Supplementary method 10: Identification of differential expressed genes (DEGs) and functional enrichment analysis**

RNAs differential expression analysis was performed by DESeq2(17) software between PFC of two different groups. Only genes with the parameter of p value below 0.05 and absolute fold change ≥ 0.58 were considered differentially expressed genes. The identified DEGs were further visualized into circle heatmap based on Chiplot(18, 19) (https://www.chiplot.online/). Next, functional enrichment analysis was performed on DEGs of each brain region between two groups of mice separately based on the Database for Annotation, Visualization and Integrated Discovery (DAVID) (20, 21). Only terms with p value under 0.05 were considered significant. The enrichment results were further visualized into chord map via R package GOplot(22).

**Supplementary method 11: Gene Set Enrichment Analysis (GSEA) and cell type specific analysis**

Gene Set Enrichment Analysis (GSEA) was conducted based on transcriptional data from PFC of WT mice and MT mice. GO and KEGG gene sets were employed in this analysis. Top 20 GO terms and KEGG pathways upregulated in MT mice were selected based on normalized enrichment score (NES). In addition, cell type-specific markers derived from several previously published single-cell RNA sequencing datasets of mouse brain cells were used to construct the brain cell type gene sets required for GSEA(23-25). All the analysis mentioned above were performed using software GSEA and MSigDB (26). (Only terms or pathways with |NES| > 1, NOM p-value < 0.05 and FDR q-value < 0.25 were considered significant.)

# Supplementary Figures and Tables

**Supplementary Table 1: The top 20 significant terms from the GSEA-KEGG pathways enrichment analysis of genes correlated with DC differences between AD patients and HCs.**

| Term | es | nes | p-val | fdr |
| --- | --- | --- | --- | --- |
| Ribosome | 0.563015 | 2.842378 | 0 | 0 |
| Staphylococcus aureus infection | 0.581009 | 2.42061 | 0 | 0 |
| Terpenoid backbone biosynthesis | 0.620078 | 2.076058 | 0 | 0 |
| Asthma | 0.667401 | 2.079991 | 0 | 0 |
| Autoimmune thyroid disease | 0.576469 | 2.106927 | 0.001 | 0.059 |
| Allograft rejection | 0.540706 | 1.960084 | 0.002 | 0.07375 |
| Graft-versus-host disease | 0.539648 | 1.93419 | 0.002 | 0.07375 |
| Steroid biosynthesis | 0.535881 | 1.697062 | 0.002 | 0.07375 |
| Proteasome | 0.52168 | 2.15192 | 0.004 | 0.107273 |
| Inflammatory bowel disease | 0.462364 | 1.863138 | 0.004 | 0.107273 |
| Fatty acid degradation | 0.447737 | 1.755023 | 0.004 | 0.107273 |
| Intestinal immune network for IgA production | 0.471903 | 1.75365 | 0.005 | 0.113462 |
| Protein export | 0.48314 | 1.700823 | 0.005 | 0.113462 |
| Propanoate metabolism | 0.463043 | 1.752357 | 0.006 | 0.126429 |
| Systemic lupus erythematosus | 0.516197 | 2.057841 | 0.007 | 0.129063 |
| PPAR signaling pathway | 0.461362 | 1.981896 | 0.007 | 0.129063 |
| Histidine metabolism | 0.442722 | 1.436994 | 0.009 | 0.140476 |
| Viral myocarditis | 0.439663 | 1.894855 | 0.01 | 0.140476 |
| Butanoate metabolism | 0.459259 | 1.626582 | 0.01 | 0.140476 |
| Porphyrin and chlorophyll metabolism | 0.435263 | 1.527786 | 0.01 | 0.140476 |

**Supplementary Table 2: The top 20 significant terms from the GSEA-Hallmark pathway enrichment analysis of genes correlated with DC differences between AD patients and HCs.**

| Term | es | nes | p-val | fdr |
| --- | --- | --- | --- | --- |
| Cholesterol Homeostasis | 0.448731 | 2.01779 | 0.001 | 0.025 |
| Reactive Oxygen Species Pathway | 0.412147 | 1.720131 | 0.001 | 0.025 |
| Myc Targets V1 | 0.381431 | 2.070906 | 0.002 | 0.03 |
| Notch Signaling | 0.415433 | 1.544485 | 0.003 | 0.03 |
| Hedgehog Signaling | 0.383363 | 1.46319 | 0.003 | 0.03 |
| Fatty Acid Metabolism | 0.430909 | 2.204872 | 0.004 | 0.033333 |
| Coagulation | 0.368683 | 1.800387 | 0.007 | 0.05 |
| Xenobiotic Metabolism | 0.389195 | 2.063882 | 0.01 | 0.061111 |
| Apical Surface | 0.3219 | 1.278455 | 0.011 | 0.061111 |
| Interferon Alpha Response | 0.29767 | 1.420204 | 0.018 | 0.09 |
| Allograft Rejection | 0.336119 | 1.744792 | 0.027 | 0.117857 |
| Glycolysis | 0.324243 | 1.739284 | 0.029 | 0.117857 |
| Bile Acid Metabolism | 0.263086 | 1.253063 | 0.032 | 0.117857 |
| Complement | 0.325573 | 1.736397 | 0.033 | 0.117857 |
| Estrogen Response Late | 0.270173 | 1.451112 | 0.038 | 0.126667 |
| Epithelial Mesenchymal Transition | 0.275996 | 1.473164 | 0.042 | 0.13125 |
| Myogenesis | 0.29274 | 1.56026 | 0.055 | 0.145 |
| Apoptosis | 0.285239 | 1.481075 | 0.055 | 0.145 |
| Oxidative Phosphorylation | 0.292826 | 1.596833 | 0.058 | 0.145 |
| Pperoxisome | 0.247479 | 1.203659 | 0.058 | 0.145 |

**Supplementary Table 3: The top 20 significant terms from the GSEA-GO cellular component pathway enrichment analysis of genes correlated with DC differences between AD and HC**

| Term | es | nes | p-val | fdr |
| --- | --- | --- | --- | --- |
| Cytosolic Large Ribosomal Subunit (GO:0022625) | 0.676035 | 2.838367 | 0 | 0 |
| Cytosolic Small Ribosomal Subunit (GO:0022627) | 0.687759 | 2.726391 | 0 | 0 |
| Large Ribosomal Subunit (GO:0015934) | 0.676035 | 2.881768 | 0 | 0 |
| Polysomal Ribosome (GO:0042788) | 0.685464 | 2.535852 | 0 | 0 |
| Small Ribosomal Subunit (GO:0015935) | 0.690723 | 2.800101 | 0 | 0 |
| MHC Protein Complex (GO:0042611) | 0.673242 | 2.223923 | 0 | 0 |
| Ribosome (GO:0005840) | 0.582106 | 2.573647 | 0.002 | 0.05925 |
| Mitochondrial Respiratory Chain Complex IV (GO:0005751) | 0.551701 | 1.751273 | 0.002 | 0.05925 |
| Multivesicular Body Membrane (GO:0032585) | 0.515199 | 1.646305 | 0.006 | 0.1422 |
| Kinetochore Microtubule (GO:0005828) | 0.457867 | 1.410511 | 0.006 | 0.1422 |
| Mitochondrial Respiratory Chain Complex I (GO:0005747) | 0.493077 | 1.997339 | 0.007 | 0.150818 |
| Anaphase-Promoting Complex (GO:0005680) | 0.501745 | 1.658349 | 0.01 | 0.1975 |
| U4/U6 X U5 tri-snRNP Complex (GO:0046540) | 0.472104 | 1.819692 | 0.011 | 0.200538 |
| U1 snRNP (GO:0005685) | 0.442285 | 1.342641 | 0.012 | 0.203143 |
| Pigment Granule (GO:0048770) | 0.490553 | 1.58639 | 0.013 | 0.2054 |
| Respiratory Chain Complex I (GO:0045271) | 0.493077 | 1.987208 | 0.016 | 0.237 |
| Spliceosomal tri-snRNP Complex (GO:0097526) | 0.472104 | 1.825528 | 0.017 | 0.237 |
| Respiratory Chain Complex IV (GO:0045277) | 0.48728 | 1.562048 | 0.018 | 0.237 |
| U5 snRNP (GO:0005682) | 0.472306 | 1.537763 | 0.019 | 0.237 |
| Heterotrimeric G-protein Complex (GO:0005834) | 0.455449 | 1.535501 | 0.025 | 0.29625 |

**Supplementary Table 4: Associations between neuroimaging phenotypes and neurotransmitter activity maps**

| 'PET Map' | 'Mean Fisher''s z (Pearson r)' | 'p_exact (spatial permutations)' |
| --- | --- | --- |
| DC differences in the LSFG | | |
| 5HT1a_WAY | 0.0944 | 0.34765 |
| 5HT1a_cumi | 0.0783 | 0.46354 |
| 5HT1b_P943 | 0.1956 | 0.066933 |
| 5HT1b_AZ | 0.0494 | 0.58741 |
| 5HT2a_ALT | 0.0725 | 0.46653 |
| 5HT2a_cimbi | 0.087 | 0.38262 |
| 5HT4 | 0.0151 | 0.89111 |
| CB1 | 0.1195 | 0.36563 |
| CBF_ASL | 0.1056 | 0.32068 |
| D1 | -0.0604 | 0.53447 |
| D2_RA | -0.1239 | 0.1998 |
| DAT | -0.2274 | 0.008991* |
| FDOPA | -0.2116 | 0.020979* |
| GABAa_C11 | -0.0592 | 0.51449 |
| GABAa_HC16 | -0.0277 | 0.82717 |
| Kappa | -0.0258 | 0.82917 |
| MU_C11 | 0.1372 | 0.34965 |
| MU_HC39 | 0.0878 | 0.62138 |
| NAT | -0.0978 | 0.3017 |
| NMDA | -0.1868 | 0.046953 |
| SERT_DASB_HC30 | -0.0907 | 0.31968 |
| SERT_MADAM | -0.0655 | 0.49251 |
| SERT_DASB_HC100 | -0.137 | 0.16084 |
| VAChT_18 | -0.1008 | 0.27173 |
| VAChT_4 | -0.177 | 0.053946 |
| VAChT_5 | -0.1468 | 0.11588 |
| mGluR5_22 | 0.0653 | 0.55844 |
| mGluR5_28 | 0.0099 | 0.92408 |
| mGluR5_73 | -0.0508 | 0.61538 |
| FC | | |
| 5HT1a_WAY | 0.0952 | 0.072927 |
| 5HT1a_cumi | 0.0033 | 0.96004 |
| 5HT1b_P943 | 0.0237 | 0.62937 |
| 5HT1b_AZ | -0.0568 | 0.15584 |
| 5HT2a_ALT | 0.1009 | 0.060939 |
| 5HT2a_cimbi | -0.0395 | 0.53447 |
| 5HT4 | -0.0847 | 0.066933 |
| CB1 | -0.0153 | 0.81918 |
| CBF_ASL | 0.0403 | 0.47253 |
| D1 | -0.0187 | 0.79421 |
| D2_RA | 0.1339 | 0.034965* |
| DAT | 0.0546 | 0.42458 |
| FDOPA | 0.0851 | 0.16484 |
| GABAa_C11 | 0.1221 | 0.015984* |
| GABAa_HC16 | 0.0621 | 0.18482 |
| Kappa | 0.0986 | 0.28372 |
| MU_C11 | -0.1359 | 0.091908 |
| MU_HC39 | -0.1089 | 0.2048 |
| NAT | 0.1891 | 0.001998* |
| NMDA | 0.0706 | 0.16184 |
| SERT_DASB_HC30 | 0.0612 | 0.34965 |
| SERT_MADAM | 0.0618 | 0.31668 |
| SERT_DASB_HC100 | 0.024 | 0.63037 |
| VAChT_18 | -0.0278 | 0.74026 |
| VAChT_4 | 0.0468 | 0.55944 |
| VAChT_5 | -0.003 | 0.97802 |
| mGluR5_22 | -0.0162 | 0.79321 |
| mGluR5_28 | 0.0411 | 0.54745 |
| mGluR5_73 | 0.1217 | 0.076923 |

**Supplementary Table 5: TOP 20 GSEA-GO terms upregulated in PFC of Tfh13-cKO AD mice compared to AD mice**

| Term | es | nes | p-val | fdr |
| --- | --- | --- | --- | --- |
| Homophilic cell adhesion via plasma membrane adhesion molecules | 0.5937386 | 2.0958645 | 0 | 0 |
| Postsynaptic density membrane | 0.55382663 | 1.9206619 | 0 | 0.070469595 |
| Cell-cell adhesion via plasma-membrane adhesion molecules | 0.5133298 | 1.8911688 | 0 | 0.08403335 |
| Antigen binding | 0.6059526 | 1.8806155 | 0 | 0.08449598 |
| Response to acetylcholine | 0.6232764 | 1.8697642 | 0 | 0.08188449 |
| Neuron projection guidance | 0.503023 | 1.852216 | 0 | 0.096846506 |
| Axon guidance | 0.503023 | 1.8343809 | 0 | 0.11399467 |
| Cellular response to acetylcholine | 0.61803025 | 1.805228 | 0 | 0.16964836 |
| Acetylcholine receptor signaling pathway | 0.6083328 | 1.7997087 | 0 | 0.16706595 |
| Cellular response to calcium ion | 0.5469479 | 1.7942426 | 0 | 0.16347201 |
| Postsynaptic specialization membrane | 0.5072216 | 1.7857867 | 0 | 0.17172551 |
| Semaphorin-plexin signaling pathway | 0.6121311 | 1.7806921 | 0 | 0.17153557 |
| Phasic smooth muscle contraction | 0.67123455 | 1.7729126 | 0.001410437 | 0.18009277 |
| Dendritic shaft | 0.55494505 | 1.7663773 | 0 | 0.18506864 |
| Inhibitory postsynaptic potential | 0.6969653 | 1.7618252 | 0 | 0.18550976 |
| Positive regulation of vascular associated smooth muscle cell migration | 0.71819323 | 1.7516705 | 0 | 0.20348659 |
| synaptic membrane | 0.4580417 | 1.7503753 | 0 | 0.19521017 |
| Heterophilic cell-cell adhesion via plasma membrane cell adhesion molecules | 0.58583 | 1.749476 | 0 | 0.18718062 |
| Alanine transport | 0.71607196 | 1.7487575 | 0.002994012 | 0.17982312 |
| Peptidoglycan muralytic activity | 0.74011207 | 1.7412704 | 0.001477105 | 0.19332784 |

**Reference**

1. Kulthanan K, Tuchinda P, Nitiyarom R, Chunharas A, Chantaphakul H, Aunhachoke K, et al. Clinical practice guidelines for the diagnosis and management of atopic dermatitis. Asian Pacific journal of allergy and immunology. 2021;39(3):145-55.

2. Ständer S. Atopic Dermatitis. N Engl J Med. 2021;384(12):1136-43.

3. Veale JF. Edinburgh Handedness Inventory - Short Form: a revised version based on confirmatory factor analysis. Laterality. 2014;19(2):164-77.

4. Chopra R, Vakharia PP, Sacotte R, Patel N, Immaneni S, White T, et al. Severity strata for Eczema Area and Severity Index (EASI), modified EASI, Scoring Atopic Dermatitis (SCORAD), objective SCORAD, Atopic Dermatitis Severity Index and body surface area in adolescents and adults with atopic dermatitis. The British journal of dermatology. 2017;177(5):1316-21.

5. Reich A, Heisig M, Phan NQ, Taneda K, Takamori K, Takeuchi S, et al. Visual analogue scale: evaluation of the instrument for the assessment of pruritus. Acta dermato-venereologica. 2012;92(5):497-501.

6. Wang W, Bian Q, Zhao Y, Li X, Wang W, Du J, et al. Reliability and validity of the Chinese version of the Patient Health Questionnaire (PHQ-9) in the general population. Gen Hosp Psychiatry. 2014;36(5):539-44.

7. Yan CG, Wang XD, Zuo XN, Zang YF. DPABI: Data Processing & Analysis for (Resting-State) Brain Imaging. Neuroinformatics. 2016;14(3):339-51.

8. Friston KJ, Williams S, Howard R, Frackowiak RS, Turner R. Movement-related effects in fMRI time-series. Magn Reson Med. 1996;35(3):346-55.

9. Zuo XN, Ehmke R, Mennes M, Imperati D, Castellanos FX, Sporns O, et al. Network centrality in the human functional connectome. Cereb Cortex. 2012;22(8):1862-75.

10. Martins D, Giacomel A, Williams SCR, Turkheimer F, Dipasquale O, Veronese M. Imaging transcriptomics: Convergent cellular, transcriptomic, and molecular neuroimaging signatures in the healthy adult human brain. Cell Rep. 2021;37(13):110173.

11. Steinhoff M, Ahmad F, Pandey A, Datsi A, AlHammadi A, Al-Khawaga S, et al. Neuroimmune communication regulating pruritus in atopic dermatitis. The Journal of Allergy and Clinical Immunology. 2022;149(6):1875-98.

12. Arnatkeviciute A, Fulcher BD, Fornito A. A practical guide to linking brain-wide gene expression and neuroimaging data. NeuroImage. 2019;189:353-67.

13. Hawrylycz MJ, Lein ES, Guillozet-Bongaarts AL, Shen EH, Ng L, Miller JA, et al. An anatomically comprehensive atlas of the adult human brain transcriptome. Nature. 2012;489(7416):391-9.

14. Markello RD, Arnatkeviciute A, Poline JB, Fulcher BD, Fornito A, Misic B. Standardizing workflows in imaging transcriptomics with the abagen toolbox. eLife. 2021;10.

15. Desikan RS, Ségonne F, Fischl B, Quinn BT, Dickerson BC, Blacker D, et al. An automated labeling system for subdividing the human cerebral cortex on MRI scans into gyral based regions of interest. NeuroImage. 2006;31(3):968-80.

16. Dukart J, Holiga S, Rullmann M, Lanzenberger R, Hawkins PCT, Mehta MA, et al. JuSpace: A tool for spatial correlation analyses of magnetic resonance imaging data with nuclear imaging derived neurotransmitter maps. Hum Brain Mapp. 2021;42(3):555-66.

17. Love MI, Huber W, Anders S. Moderated estimation of fold change and dispersion for RNA-seq data with DESeq2. Genome Biol. 2014;15(12):550.

18. Li X, Li J, Zhao Q, Qiao L, Wang L, Yu C. Physiological, biochemical, and genomic elucidation of the Ensifer adhaerens M8 strain with simultaneous arsenic oxidation and chromium reduction. Journal of Hazardous Materials. 2023;441:129862.

19. Ji X, Tang J, Zhang J. Effects of Salt Stress on the Morphology, Growth and Physiological Parameters of Juglansmicrocarpa L. Seedlings. Plants (Basel, Switzerland). 2022;11(18):2381.

20. Sherman BT, Hao M, Qiu J, Jiao X, Baseler MW, Lane HC, et al. DAVID: a web server for functional enrichment analysis and functional annotation of gene lists (2021 update). Nucleic Acids Research. 2022;50(W1):W216-W21.

21. Huang DW, Sherman BT, Lempicki RA. Systematic and integrative analysis of large gene lists using DAVID bioinformatics resources. Nature Protocols. 2009;4(1):44-57.

22. Walter W, Sanchez-Cabo F, Ricote M. GOplot: an R package for visually combining expression data with functional analysis. Bioinformatics. 2015;31(17):2912-4.

23. Wu YE, Pan L, Zuo Y, Li X, Hong W. Detecting Activated Cell Populations Using Single-Cell RNA-Seq. Neuron. 2017;96(2):313-29 e6.

24. Rosenberg AB, Roco CM, Muscat RA, Kuchina A, Sample P, Yao Z, et al. Single-cell profiling of the developing mouse brain and spinal cord with split-pool barcoding. Science. 2018;360(6385):176-82.

25. Paul A, Crow M, Raudales R, He M, Gillis J, Huang ZJ. Transcriptional Architecture of Synaptic Communication Delineates GABAergic Neuron Identity. Cell. 2017;171(3):522-39 e20.

26. Subramanian A, Tamayo P, Mootha VK, Mukherjee S, Ebert BL, Gillette MA, et al. Gene set enrichment analysis: a knowledge-based approach for interpreting genome-wide expression profiles. Proc Natl Acad Sci U S A. 2005;102(43):15545-50.
