# Supplementary figures and images for: Cross-species transcriptomic evidence for peripheral–central immune crosstalk in atopic dermatitis
Source: Front Immunol. 2026 Jun 26;17:1790710. doi: 10.3389/fimmu.2026.1790710 (PMC13349758; doi:10.3389/fimmu.2026.1790710)

## Slide 1
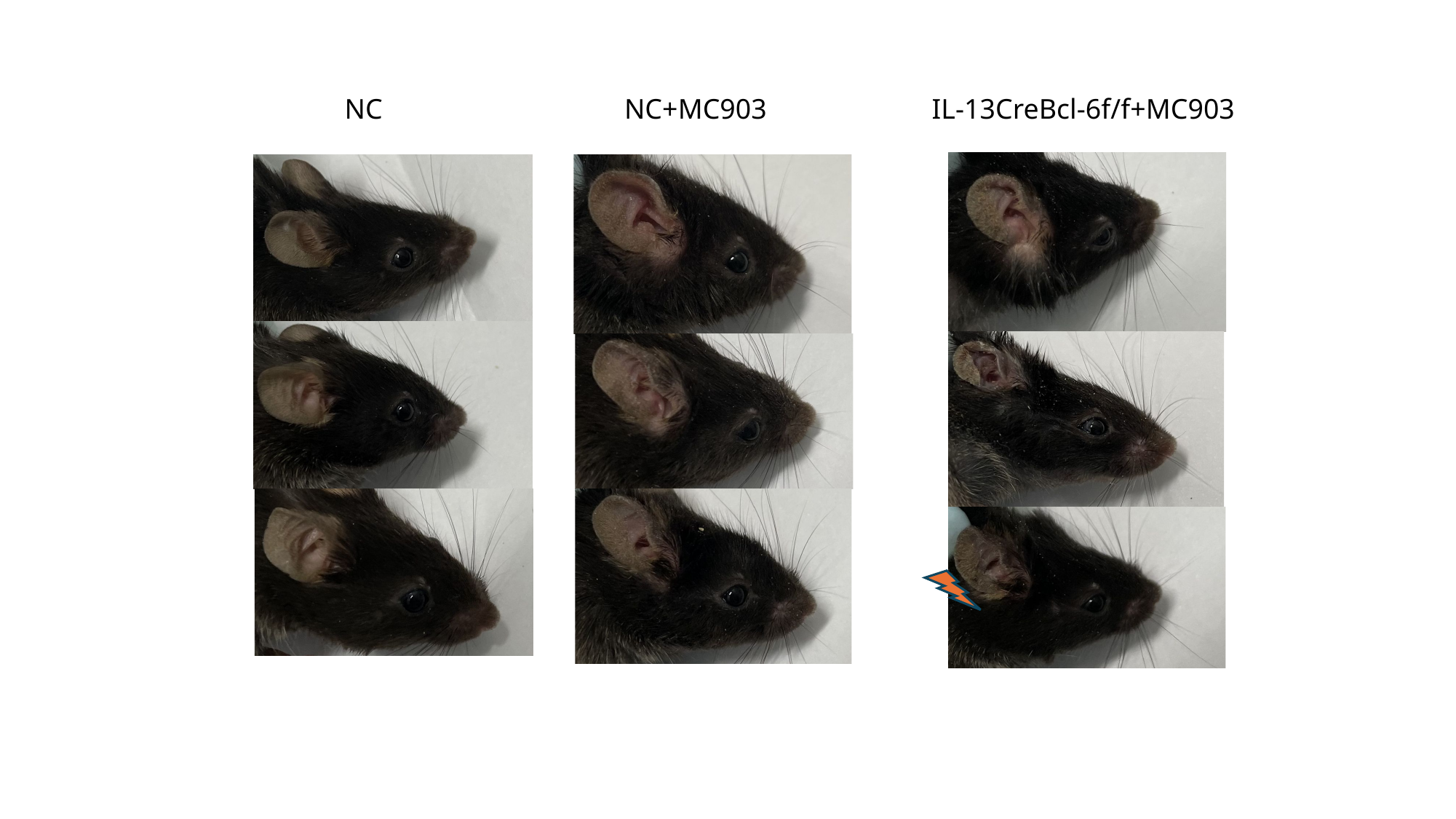

NC
NC+MC903
IL-13CreBcl-6f/f+MC903

## Slide 2
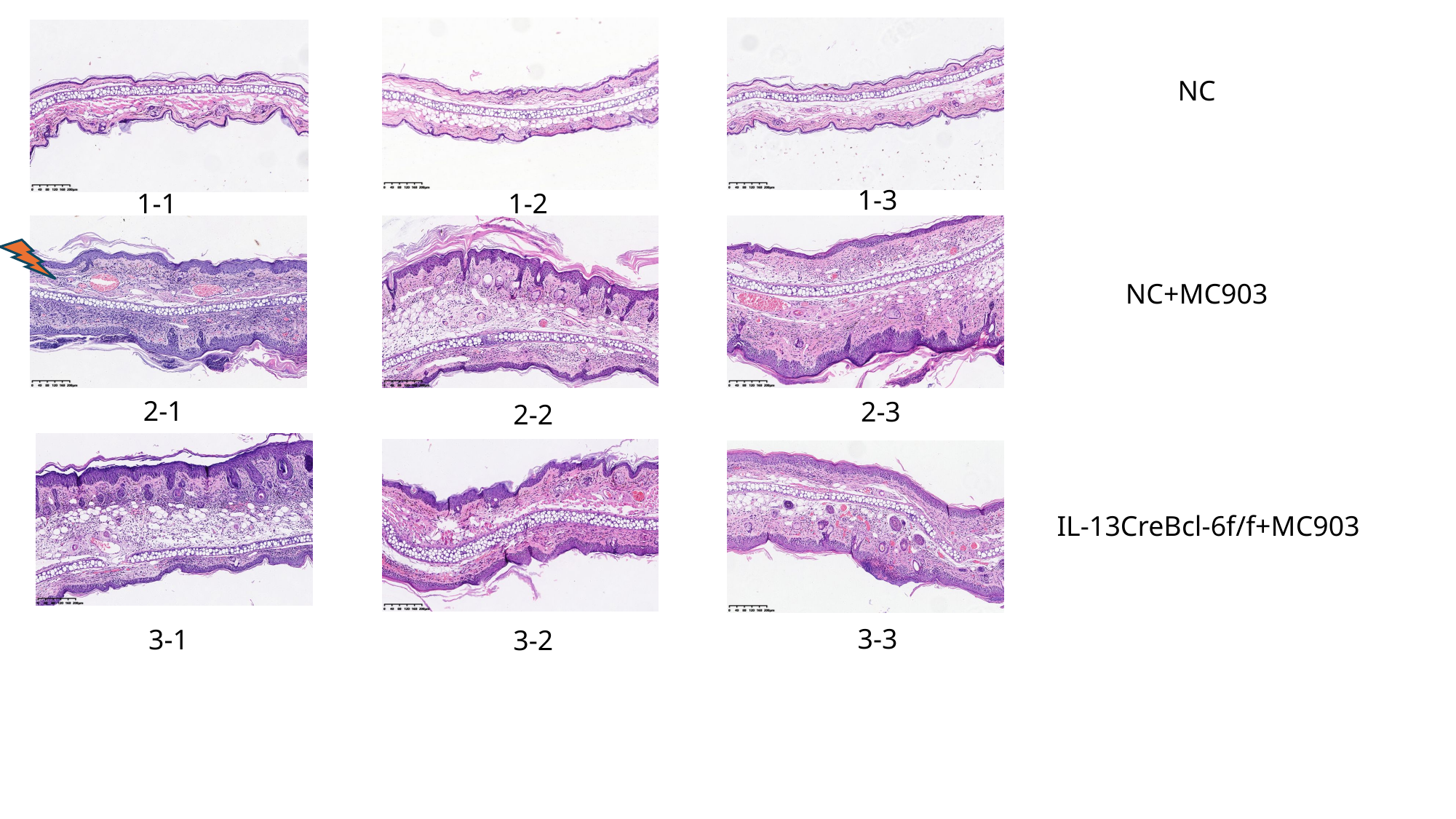

NC
1-3
1-1
1-2
NC+MC903
2-1
2-3
2-2
IL-13CreBcl-6f/f+MC903
3-3
3-1
3-2

Supplement: Supplementary file 2 [file Supplementaryfile1.pptx]
